# Changes in enzymatic activity and oxidative stress in honeybees kept in the apiary and laboratory conditions during the course of nosemosis

**DOI:** 10.1371/journal.pone.0317384

**Published:** 2025-01-15

**Authors:** Magdalena Kunat-Budzyńska, Emilia Łabuć, Aneta A. Ptaszyńska

**Affiliations:** 1 Department of Immunobiology, Institute of Biological Sciences, Faculty of Biology and Biotechnology, Maria Curie-Skłodowska University, Lublin, Poland; 2 Laboratory of Bioinformatics and Biostatistics, Institute of Biological Sciences, Faculty of Biology and Biotechnology, Maria Curie-Skłodowska University, Lublin, Poland; Gomal University, PAKISTAN

## Abstract

The aim of this study was to investigate the changes in the level of oxidative stress and lysozyme-like and phenoloxidase (PO) activity under the influence of nosemosis. Honeybees were kept in natural (apiary) and artificial (laboratory) conditions. In this study, it was shown for the first time that honeybees kept in apiaries have higher levels and activity of the studied parameters than honeybees kept in the laboratory. The greatest difference was noted in the case of PO activity in 28-day-old infected honeybees in May, when the activity was 32.3 times higher in honeybees kept in the apiary than in the laboratory, suggesting that environmental conditions have a significant influence on the immune response of honeybees. Simultaneously, the apiary conditions resulted in higher level of oxidative stress, indicating lower effectiveness of antioxidative mechanisms. Additional nosemosis infection increased the level of oxidative stress as well as lysozyme and PO activities. In July, in 28-day-old infected honeybees kept in laboratory, the highest increase in PO activity (by 10.79 fold) was detected compared to healthy honeybees. This may indicate that infection causes a decrease in the effectiveness of primarily antioxidant mechanisms, whereas immune mechanisms are still activated during infection. Another interesting factor is the age of the honeybees. It was found that in the summer months (June, July) the lysozyme-like and PO activities increased with age, while in the case of oxidative stress the opposite trend was observed, suggesting better effectiveness of both immune and antioxidant mechanisms. Another important element is seasonality, which significantly affected only the lysozyme-like activity. It was found that in July in all the groups studied this activity was higher than in the other months. The results allow us to better understand the mechanisms of honeybee immunity, which are constantly being studied due to the complex social structure created by these insects. Our research emphasizes that honeybee immunity is dynamic and depends on a number of factors, such as environment, age, season or the presence of pathogens.

## 1. Introduction

One of the most important pollinators on Earth is the honeybee *Apis mellifera* L., which influences the preservation of biodiversity and is a bioindicator associated with the control of the state of environment [1, 2]. The homeostasis of honeybees is very often disturbed by different stressors. These factors can be divided into two groups, i.e. biotic and abiotic factors. Biotic factors include pathogens as: bacteria (*Paenibacillus larvae*, *Spiroplasma apis*), fungi (*Aspergillus flavus*, *Ascosphaera apis*), viruses (*Deformed wing virus*, *Black queen cell virus*) and parasites (*Vairimorpha ceranae*, *Varroa destructor*). Abiotic factors include temperature, climate change, the use of pesticides, malnutrition, the presence of heavy metals and radiation [3–5]. All of these factors lead to oxidative stress. Oxidative stress results from an imbalance between the generation of reactive oxygen species (ROS) and antioxidant defense mechanisms [2, 4, 6–8]. Reactive oxygen species affect protein oxidation, DNA and RNA fragmentation and also cause lipid peroxidation. Oxidative stress leads to aging processes, damage to macromolecular structures, apoptosis, carcinogenesis, and physiological disorders that disrupt biological processes [9–12].

To counteract the harmful effects of oxidative stress, organisms have developed defense mechanisms based on the action of enzymatic and non-enzymatic antioxidants [4]. Examples of antioxidant enzymes include catalase and glutathione S-transferase [6, 10]. However, non-enzymatic factors include, among others, vitamins C and E, glutathione, albumin, and uric acid [10]. These factors provide primary protection against oxidative stress that may occur in honeybees following infection with *Vairimorpha ceranae* (previously *Nosema ceranae*) [4, 6, 7, 11].

Increased oxygen demand in foraging honeybees leads to oxidative stress, but this condition can be alleviated by increased regulation of proteins, such as Hsp70 in the flight muscles, and the activity of antioxidant enzymes such as catalase and superoxide dismutase [13]. It has been observed that the flying ability of worker honeybees declines with age as a result of damage to the flight muscles under the influence of oxidative stress. Furthermore, a decrease in antioxidant activity has been found in the muscles of forager honeybees and nurse honeybees [14].

One of the stress factors is the presence of intracellular parasites ‐ microsporidia of the *Vairimorpha* (previously *Nosema*) genus, the causative agent of nosemosis. In Poland, nosemosis is caused by two species, i.e. *Vairimorpha ceranae* and *V*. *apis*, which are responsible for reducing the number of honeybees in apiaries [15, 16]. Nosemosis causes a permanent and progressive degradation of the intestinal epithelium, which may result in disturbances in the functioning of the digestive tract [11, 17, 18]. In addition, nosemosis reduces the host’s energy resources, such as ATP and carbohydrates, and disrupts amino acid and protein metabolism. *Vairimorpha ceranae* infection has also been shown to reduce protein levels in the midgut [11, 19]. As a result of infection, the hypopharyngeal glands, which are responsible for the production of royal jelly and glucosidase III, disappear [16, 20]. *Vairimorpha* spp. infection in honeybees also causes memory problems, a weakened immune response, e.g. decreased expression of genes encoding defense peptides, and energy and oxidative stress [7, 11, 21].

The presence of other stressors, including pathogens and pesticide use, increases the host susceptibility to infection with *V*. *ceranae* [7]. As a result of *V*. *ceranae* infection, the hormonal balance is disturbed, i.e. there are changes in the levels of vitellogenin and juvenile hormone III [16]. It has been shown that, under the influence of infection, the level of vitellogenin increases in queen honeybees and younger honeybees, while an increase in the level of juvenile hormone was observed in worker honeybees, which may consequently accelerate age-related tasks, i.e. the division of honeybees according to the type of work performed in the honeybee colony [16, 22].

To fight pathogens, insects have a highly developed innate immune system, which consists of a cellular and humoral response. The main mechanisms of the cellular response are phagocytosis, nodulation, and encapsulation [23, 24]. The humoral response consists of several processes, such as hemolymph coagulation, activation of proteolytic cascades involved in the melanization process, and induction of the synthesis of defense peptides that are directly involved in fighting infection [23, 24]. Depending on the type of attacking pathogen, insects synthesize different defense peptides that, at the time of infection, enter the hemolymph from the fat body and some hemocytes [23, 24].

The honeybee’s innate immune system uses pattern recognition receptors (PRRs) that interact with pathogen-associated molecular patterns (PAMPs) to stimulate different immune signaling pathways, such as IMD (immune deficiency), JNK (c-Jun N-terminal kinase), and JAK/STAT (Janus kinase/signal transducers and activators of transcription), depending on the pathogen recognized [25]. In addition, honeybees have immunity linked to the hygienic behavior of the honeybee family, which involves cleaning the honeycombs and keeping the hive clean by removing dead brood. Thanks to this behavior, honeybees inhibit the development of various diseases in the honeybee colony [23, 25, 26].

One of the immune proteins involved in the humoral response is lysozyme. Two classes of lysozyme have been discovered in insects: type c and type i [27, 28]. In the case of honeybees, three genes encoding lysozyme have been found: two encoding c-type lysozymes and one encoding i-type lysozyme [28]. The main function of lysozyme is to destroy the cell wall peptidoglycan mainly of Gram-positive bacteria. In the case of Gram-negative bacteria, the mechanism of lysozyme action is not fully understood [26]. Lysozyme is an indicator of immunity; hence, it is often used in scientific research to assess the condition/immunity of the honeybee colony. In insects, low levels of lysozyme can be constitutively present in the immune-competent tissues, e.g. in hemolymph. Moreover, in many insects, including *A*. *mellifera*, an expression of lysozyme genes is induced upon systemic infection and after injection of cell wall components of bacteria and fungi [25, 29–31].

Another important enzyme of the immune system in insects is phenoloxidase. Phenoloxidase (PO) is formed in insects via the activation of its proenzyme (zymogen) called prophenoloxidase (proPO) in response to various stimuli, including injuries, mechanical stimuli, and various types of chemicals [32–34]. The activation of the serine protease cascade leads to the conversion of proPO to the active form of PO through a process of limited proteolysis [32–35]. PO is the key enzyme responsible for the initiation of the melanization process, often involved in encapsulation and nodulation [36, 37].

Since many studies are conducted in laboratory conditions, the aim of this research was to check whether the various conditions, i.e. natural (apiary) and laboratory, honeybee age, and seasonality affect the level of oxidative stress and enzymatic activity in healthy and *Vairimorpha* spp. infected honeybees. The levels of total ROS/RNS and the lysozyme-like and PO activities were compared in healthy and infected 1-day-old, 19-day-old, and 28-day-old honeybees kept in the laboratory and in the apiary.

## 2. Materials and methods

### 2.1. Collection of honeybees for experiments

Buckfast honeybees were collected from a local beekeeper (Łuszczów Drugi, Poland, Lublin Voivodeship, 51°17′36″N 22°45′13″E) in May, June, July, August, and September 2023. Two honeybee colonies were selected for the experiments: a healthy colony (group 1) and honeybees infected with nosemosis (group 2). The colonies of sister queen bees were chosen for the study to minimize genetic differences between healthy and infected colonies. The frames with brood were taken from each colony each month and placed in two separate incubators (temperature 34.5°C; humidity 85%) until the honeybees emerged. One-day-old honeybees were divided into four groups of 500 honeybees each:

- 1L – healthy honeybees kept in the laboratory
- 2L – *V*. *ceranae*-infected honeybees kept in the laboratory
- 1A – healthy honeybees kept in the apiary
- 2A – *V*. *ceranae*-infected honeybees kept in the apiary

**Fig 1:**
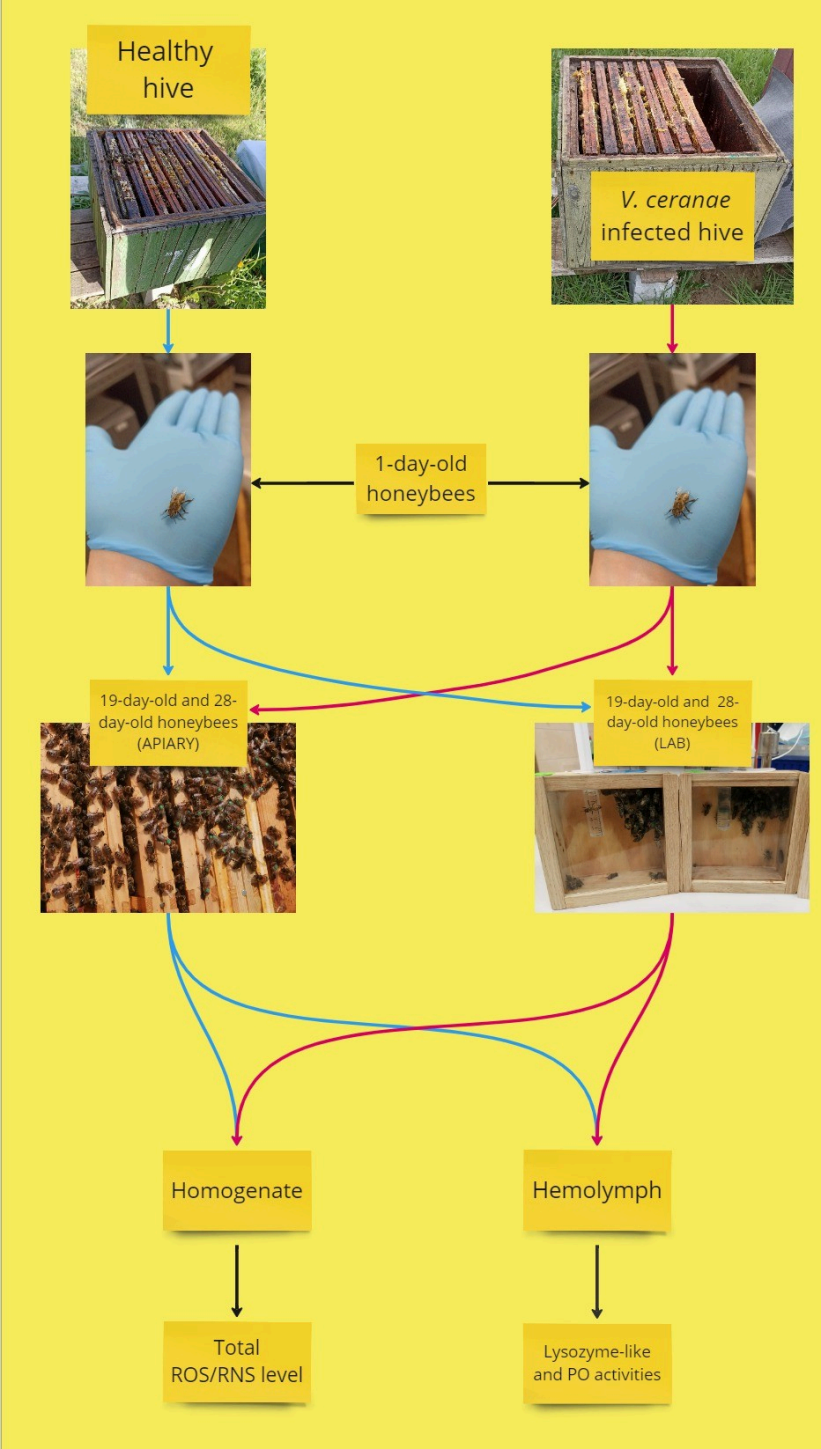
Illustrative diagram of the experiment. The red lines indicate infected honeybees and the blue lines indicate healthy honeybees.

Healthy honeybees (group 1L) and infected honeybees (group 2L) were placed in special wooden cages equipped with a hole through which a syringe with food was administered and a hole for air supply. Before the honeybees were settled, the wooden cages were heated with gas burner for disinfection. These honeybees were placed in the laboratory in appropriate humidity (H = 65%) in complete darkness and fed with pure sucrose-water syrup (1:1 *w/v*) without spores all the time (Fig 1) [38–40]. In order to ensure appropriate conditions for the development of nosemosis, the honeybees in the laboratory were kept at a constant temperature (25°C) [41]. To maintain a similar level of nosemosis infection in the apiary and in the laboratory, an infectious syrup that was administered to honeybees in the laboratory from group 2L was prepared. For this purpose, several dozen foragers were collected from the infected hive in the apiary (group 2A) each month, the honeybee abdomens have been ground in a mortar in 40% sugar syrup and filtered through gauze. Subsequently, microscopic preparations were made and the number of spores was counted under an Olympus BX 61 light microscope using a hemocytometer (Bürker chamber). The filtrates prepared in May, June, July, August, and September contained, respectively, 4.1, 2.7, 2.0, 1.1, and 0.18 × 10^6^ *V*. *ceranae* spores/mL. The filtrates were fed to the honeybees for 5 days from the moment of emergence [42, 43].

Healthy honeybees (group 1A) and infected honeybees (2A) were marked with a special marker and returned to their colony in the apiary (Fig 1).

Honeybees were collected for analysis in the following variants: 1-day-old, 19-day-old, and 28-day-old after emergence due to their functions in the hive.

### 2.2. Hemolymph collection from honeybees

From each experimental variant, hemolymph was collected in sterile conditions from the antennae of five individuals into cooled, sterile Eppendorf tubes in three biological repetitions [44]. The hemolymph was then centrifuged to remove hemocytes in the following conditions: 200×g for 5 min. at 4°C, the supernatant was collected and centrifuged again at 20,000×g for 15 min. After centrifugation, the hemolymph was directly used for testing lysozyme-like activity or was kept at -20°C for the determination of PO activity [45] (Fig 1).

### 2.3. Determination of phenoloxidase activity

The determination of phenoloxidase activity was performed according to the method described by Ptaszyńska et al. [45]. In order to determine the PO activity in each variant in three biological replicates, the hemolymph was diluted twice with Tris-buffered saline (TBS) consisting of 50 mM Tris-HCl, pH 6.8, and 1 mM NaCl. Then, 2 μL of diluted hemolymph was combined with 18 μL of TBS supplemented with 5 mM CaCl_2_ in the wells of a 96-well plate and incubated for 20 minutes at room temperature. After incubation, 180 μL of a 2 mM L-dihydroxyphenylalanine (L-DOPA) solution in 50 mM sodium phosphate buffer (pH 6.5) was added. The spectrophotometric determination of PO activity was based on the amount of melanin produced by measuring the absorbance at 490 nm over a period of 60 min, with measurements every 15 min, using a microplate reader (Benchmark Plus Microplate Reader, Bio-Rad, USA) (Fig 1).

### 2.4. Determination of lysozyme-like activity

The determination of lysozyme-like activity in honeybee hemolymph was performed by radial diffusion assay using 1% agarose plates containing lyophilized *Micrococcus lysodeikticus* (M3770; Sigma) according to the method described by Andrejko et al. [46] and Ptaszyńska et al. [47]. Seven μL of undiluted hemolymph was added to each well in three biological replicates, and then the plates were incubated at 28°C for 24 hours. After incubation, the diameters of peptidoglycan digestion zones were measured. Lysozyme activity was calculated using a calibration curve developed with egg-white lysozyme (EWL) (Sigma; EC 3.2.1.17), and the results were expressed in μg/mL EWL (Fig 1).

### 2.5. Measurement of reactive oxygen species (ROS) and reactive nitrogen species (RNS)

The oxidative stress analysis was performed by measuring the levels of total reactive oxygen species (ROS) and total reactive nitrogen species (RNS) using the commercially available OxiSelect™ In vitro ROS/RNS Assay Kit (Green Fluorescence) (Prospecta, Warsaw, Poland). For this purpose, 5 whole individuals (pooled) were homogenized in phosphate buffered saline (PBS, pH 7.4) using a glass homogenizer in sterile conditions. The tissue homogenate was centrifuged at 10,000×g for 5 min at 4°C. After centrifugation, the supernatant was analyzed according to the manufacturer`s protocol. Fluorescence was measured at an excitation wavelength of 480 nm and an emission wavelength of 530 nm using a microplate reader (Multimode Plate Readers, PerkinElmer, USA). The level of ROS and RNS was calculated from the standard curve for 2’,7’-dichlorodihydrofluorescein (DCF). The analyses were performed for all the tested groups in three biological replicates [48].

### 2.6. Determination of *Vairimorpha* spp. infection by Polymerase Chain Reaction (PCR)

The polymerase chain reaction (PCR) was used to determine the presence of *Vairimorpha* spp. infection in the tested groups. For this purpose, DNA was isolated from honeybee abdomens (three pooled abdomens) from each tested group in three biological replicates according to the manufacturer’s protocol (EurX, Gdańsk). The isolated DNA was stored at -20°C for further analysis. Pathogens were identified by PCR using primers specific for [49, 50]:

- *V*. *ceranae*: FOR 5′-CGGCGACGATGTGATATGAAAATATTAA-3′; REV 5′-CCCGGTCATTCTCAAACAAAAAACCG-3′;
- *V*. *apis*: FOR 5′-GGGGGCATGTCTTTGACGTACTATGTA-3′; REV 5′- GGGGGGCGTTTAAAATGTGAAACAACTATG-3′.

The PCR was performed using the following conditions: initial denaturation at 95°C (3 min), and 35 cycles of (95°C for 1 min, 46°C for 1 min, 72°C for 1 min), and a final extension cycle at 72°C (10 min) [49, 50]. The PCR products were subjected to electrophoretic separation (2% agarose gel). The image of the gel was visualized by the ChemiDoc™ Imaging System (Bio-Rad, USA). The size of the bands for *V*. *apis* (321 bp) and *V*. *ceranae* (218 bp) were compared with the DNA size standard (GeneRuler 100 bp DNA Ladder, ThermoFischer Scientific, Waltham, MA, USA).

### 2.7. Statistical analysis

The analyses were carried out using Python version 3.11 with the math, pandas, polars, openpyxl, and scipy libraries (stats package). The charts were generated using the plotly package. To check that the data met the assumption of normality of distribution, a Shapiro-Wilk test was performed for each month, for each age category of honeybees, for each group: L (honeybees kept in the laboratory) and A (honeybees from the apiary), and for each group with: 1 (healthy honeybees) and 2 (infected honeybees). In most cases, at the significance level α = 0.1, the null hypothesis that the data come from a normal distribution cannot be rejected. In order to test the homogeneity of the variance between the studied groups, a Levene test was performed at a significance level of α = 0.05; the hypothesis of equality of variance across the groups was rejected. Consequently, one-sided Welch’s t tests were used in further analyses.

#### 2.7.1. Comparison of the tested parameters in honeybees kept in the apiary and in the laboratory

**Fig 2:**
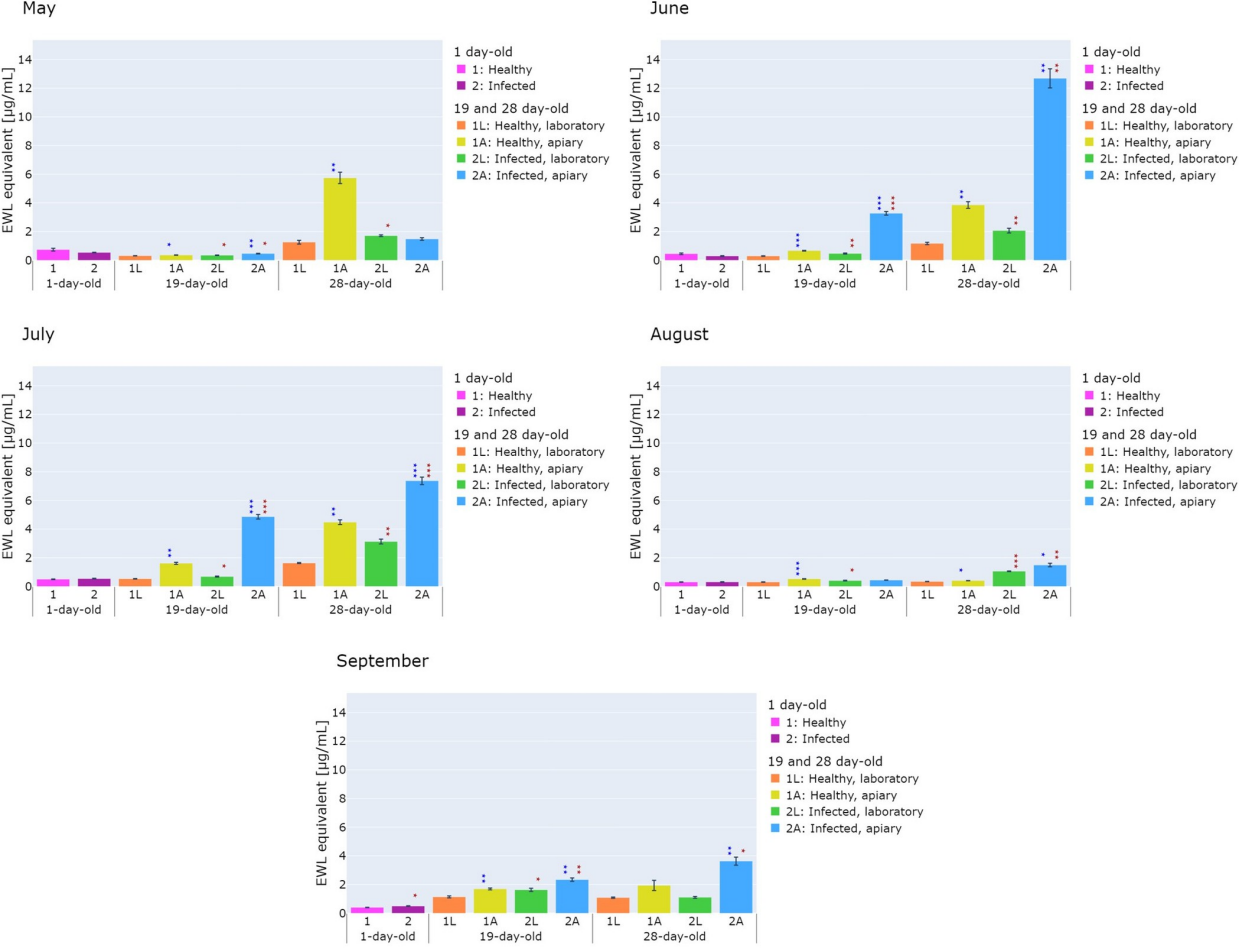
Lysozyme-like activity in the hemolymph of healthy (1L, 1A) and *V*. *ceranae*-infected (2L, 2A) honeybees collected in 3 time variants in May, June, July, August, and September and kept in the laboratory (1L, 2L) or the apiary (1A, 2A) calculated as the equivalent of EWL activity (μg/mL). Statistically significant differences in the level of lysozyme-like activity between healthy and infected honeybees are marked in red: ★ 0.01 ≤ p-value < 0.05, ★★ 0.001 ≤ p-value < 0.01, ★★★ p-value < 0.001, while differences in the level of lysozyme-like activity between honeybees kept in the laboratory and in the apiary are marked in blue ★ 0.01 ≤ p-value < 0.05, ★★ 0.001 ≤ p-value < 0.01, ★★★ p-value < 0.001.

**Fig 3:**
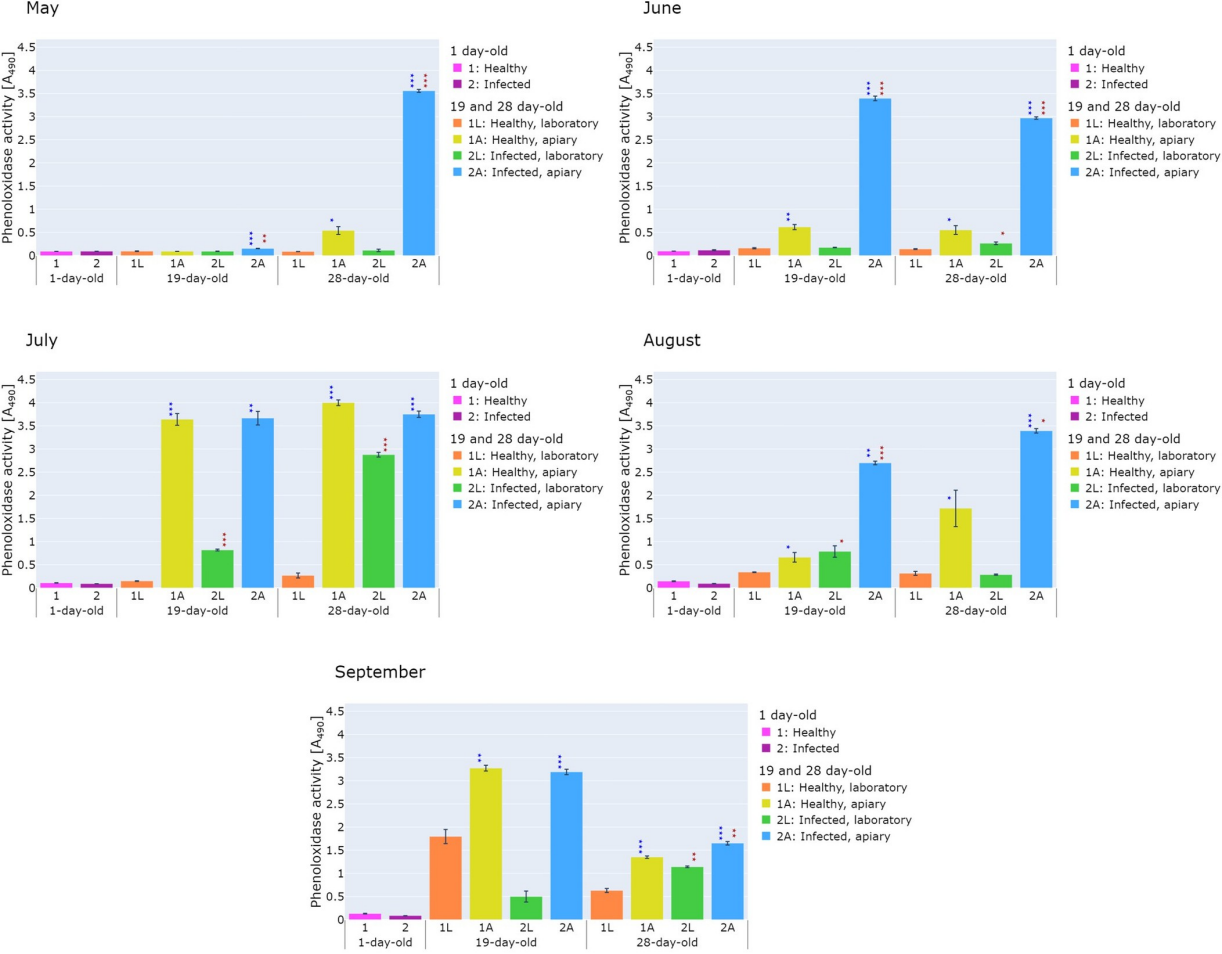
Phenoloxidase activity in the hemolymph of healthy (1L, 1A) and *V*. *ceranae*-infected (2L, 2A) honeybees collected in 3 time variants in May, June, July, August, and September and kept in the laboratory (1L, 2L) or the apiary (1A, 2A). Statistically significant differences in the level of PO activity between healthy and infected honeybees are marked in red: ★ 0.01 ≤ p-value < 0.05, ★★ 0.001 ≤ p-value < 0.01, ★★★ p-value < 0.001, while differences in the level of PO activity between honeybees kept in the laboratory and in the apiary are marked in blue ★ 0.01 ≤ p-value < 0.05, ★★ 0.001 ≤ p-value < 0.01, ★★★ p-value < 0.001.

**Fig 4:**
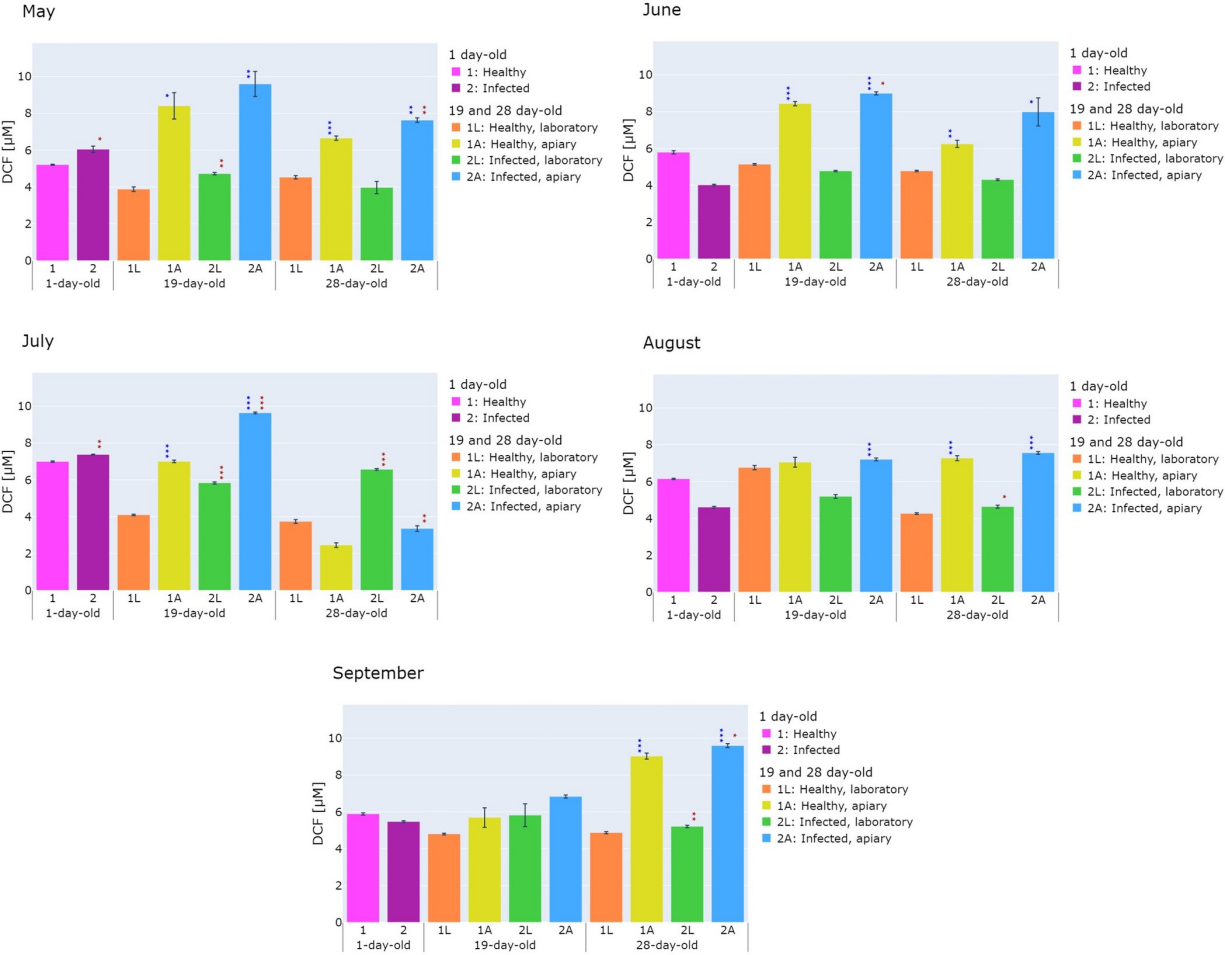
Total ROS/RNS level in the homogenates of healthy (1L, 1A) and *V*. *ceranae*-infected (2L, 2A) honeybees collected in 3 time variants in May, June, July, August, and September and kept in the laboratory (1L, 2L) or the apiary (1A, 2A). The level of ROS/RNS was read from the DCF standard curve (μM). Statistically significant differences in the level of total ROS/RNS between healthy and infected honeybees are marked in red ★ 0.01 ≤ p-value < 0.05, ★★ 0.001 ≤ p-value < 0.01, ★★★ p-value < 0.001, while differences in the level of total ROS/RNS between honeybees kept in the laboratory and in the apiary are marked in blue ★ 0.01 ≤ p-value < 0.05, ★★ 0.001 ≤ p-value < 0.01, ★★★ p-value < 0.001.

In order to compare lysozyme-like activity, PO activity, and oxidative stress levels in groups L and A, one-sided t-Welch tests were performed for each month: May, June, July, August, and September, for each honeybee age category: 1-day-old, 19-day-old, and 28-day-old, and groups 1 (1L compared to 1A) and 2 (2L compared to 2A). The null hypothesis that the lysozyme-like activity, PO activity, and oxidative stress levels in group L honeybees are higher than these parameters in group A honeybees was tested. The results, together with the significance of the tests (blue asterisks), are presented as bar graphs in the figures (Figs 2–4).

#### 2.7.2. Comparison of the tested parameters in healthy and infected honeybees

To compare lysozyme-like activity, PO activity, and oxidative stress levels in groups 1 and 2, one-sided t-Welch tests were performed for each month: May, June, July, August, and September. For 1-day-old honeybees, healthy bees (group 1) and infected bees (group 2) were compared. For 19-day-old and 28-day-old honeybees kept in the apiary: healthy group 1A was compared to infected group 2A and for honeybees kept in the laboratory: healthy group 1L was compared to infected group 2L. The null hypothesis that the lysozyme-like activity, PO activity, and oxidative stress levels in group 1 honeybees are higher than these parameters in group 2 honeybees was tested. The results, together with the significance of the tests (red asterisks), are presented as bar graphs in the figures (Figs 2–4).

#### 2.7.3. Comparison of the tested parameters depending on seasonality

In the case of healthy (1A and 1L) and *Vairimorpha*-infected (2A, 2L) honeybee colonies, a one-sided t-Welch test was performed for individual months with the null hypothesis: the level of oxidative stress and the activities of phenoloxidase and lysozyme were higher in the spring and summer months (May, June, July) compared to the months of late summer and autumn (August, September).

## 3. Results

The results of the experiments on parameters related to the humoral response, i.e. lysozyme-like activity, PO activity, and oxidative stress levels, are presented in Figs 2–4. The results indicated that the parameters studied were influenced by the apiary/laboratory conditions and nosemosis. Moreover, it was shown that seasonality affects the activity of the lysozyme-like, but no significant differences were observed in the activity of phenoloxidase and oxidative stress level.

### 3.1. Lysozyme-like and phenoloxidase activity

The natural/laboratory conditions and nosemosis had a significant effect on the level of lysozyme-like activity. The statistical analysis showed that the lysozyme-like activity in most of the healthy and infected 19-day-old and 28-day-old honeybees collected from May to September and kept in the apiary was significantly higher than the lysozyme-like activity in honeybees that were kept in the laboratory. The exceptions were the infected: 28-day-old (May) and 19-day-old (August) and healthy 28-day-old (September) honeybees.

Moreover, it was noted that the lysozyme-like activity in most of the infected 19-day-old and 28-day-old honeybees collected from May to September and kept in the apiary and in the laboratory was significantly higher than the lysozyme-like activity in the healthy honeybees (Fig 2). The greater difference was found in June between 19-day-old infected honeybees and healthy honeybees kept in the apiary (4.93-fold, p<0.01). The exceptions were the 28-day-old (May), 19-day-old (August) honeybees kept in the apiary and 28-day-old honeybees kept in the laboratory (September). However, in the healthy and infected 1-day-old honeybees, the lysozyme-like activity remained at a similar low level (Fig 2).

For the effect of apiary/laboratory conditions, the greatest difference in lysozyme-like activity was observed in June and July in 19-day-old infected honeybees, where the activity was about 7-fold (June, p < 0.01) and 7.1-fold (July, p < 0.001) higher for group 2A than for group 2L.

Additionally, it was found that in July the lysozyme-like activity was higher in all tested groups (1A, 1L, 2A, 2L) compared to other months analyzed. In 28-day-old honeybees, activity was higher in July compared to September and in June compared to August and September (with the exception of 28-day-old healthy honeybees kept in the laboratory).

As in the case of lysozyme-like activity, the natural/laboratory conditions had a significant impact on the level of PO activity. The statistical analysis showed that the PO activity in most of the healthy and infected 19-day-old and 28-day-old honeybees collected from May to September and kept in the apiary was significantly higher than the PO activity in the laboratory honeybees (Fig 3). The exception were the healthy 19-day-old honeybees (May). The greatest differences in the PO activity were observed in the 28-day-old honeybees from group 2A (May) and the 19-day-old honeybees from group 1A (July) in which the PO activity was 32.3-fold (p < 0.001) and 24.8-fold (p < 0.001) higher, respectively, compared to the 28-day-old honeybees from group 2L and the 19-day-old honeybees from group 1L (Fig 3).

It was observed that in the case of infection, the largest difference (10.79-fold, p < 0.001) was found in July in 28-day-old honeybees from group 2L compared to 28-day-old honeybees from group 1L (Fig 3).

### 3.2. Oxidative stress level

Based on the experimental results, it was found that the level of oxidative stress in most of the healthy and infected 19-day-old and 28-day-old honeybees collected from May to September and kept in the apiary was significantly higher than the level of oxidative stress in honeybees kept in the laboratory (Fig 4). The exceptions were the 28-day-old honeybees (July), 19-day-old honeybees (September) and the healthy 19-day-old honeybees (August).

Additionally, the level of oxidative stress was higher in most of the infected honeybees than in the healthy honeybees in July. A slight increase in oxidative stress in the laboratory was observed in the healthy 19-day-old (June, p < 0.05) and 28-day-old honeybees (May, p < 0.01), compared to the infected honeybees.

In the case of the natural/laboratory conditions, the greatest difference in the total ROS/RNS level was noted in the healthy 19-day-old honeybees kept in the apiary, i.e. it was 2.2-fold (p < 0.05) greater than in the healthy 19-day-old honeybees kept in the laboratory (May). However, in the case of the infection, the level of oxidative stress was 1.75-fold (p < 0.001) higher in the 28-day-old honeybees from group 2L than in the 28-day-old honeybees from group 1L in July (Fig 4).

### 3.3. Determination of *Vairimorpha* spp. infection by Polymerase Chain Reaction (PCR)

Based on the results obtained, it was concluded that there were no *V*. *ceranae* and *V*. *apis* spores in the hive from group 1. However, in the second hive (group 2), the PCR method yielded a product of 219 bp, which indicates infection (nosemosis) caused by *V*. *ceranae* in all the tested time variants and months, except for the 1-day-old honeybees collected in August (S1–S5 Figs and S1 Table in S1 File).

## 4. Discussion

In the present study, the lysozyme-like activity, PO activity, and the level of oxidative stress were compared for the first time between healthy and *Vairimorpha*-infected honeybees kept in the laboratory and natural (apiary) conditions.

Lysozyme is one of the tested parameters constituting a part of the insect humoral response. It is synthesized mainly in the fat body and has antifungal and antibacterial activity [51, 52]. Lysozyme can also influence the expression of various antimicrobial peptides [53–55] and act synergistically with these peptides [56]. The results of our experiments indicated that nosemosis infection causes an increase in lysozyme-like activity (Fig 2). Our results are consistent with a study conducted by Sinpoo et al. [57], who showed that, during *V*. *ceranae* infection, there is an increase in lysozyme gene expression leading to increased lysozyme synthesis, which is manifested by an increase in its activity. Also Doublet et al. [30] proved that *V*. *ceranae* infection influences the induction of the expression of genes encoding lysozyme-related proteins. It is important to mention here that the mechanism of lysozyme bactericidal activity has been thoroughly investigated, while the mechanisms of its fungicidal and fungistatic activity are still under investigation [58]. Research conducted by Callewaert and Michielis [59] showed that some c-type and i-type lysozymes can be classified as chitinolytic enzymes able to hydrolyze the β-1,4-glycosidic bonds in N-acetylglucosamine polymers. The presence of chitin coats was demonstrated in *Vairimorpha* spp. spores, which helps maintain spore rigidity and increases stress resistance in this pathogen [60]. Therefore, the chitin spore coat may be supposed being a target for enzymatic chitinolytic activity of insect lysozyme. On the other hand, Ptaszyńska et al. [45] investigated the effect of commercial prebiotics on the development of nosemosis, the lysozyme-like activity, and the composition of the yeast microflora in honeybees. The researchers observed that the lysozyme-like activity after infection with *Vairimorpha* spp. remained at a low level, similar to the lysozyme-like activity in the healthy honeybees. Similar conclusions were previously reached by Antúnez et al. [54], who observed unchanged expression of lysozyme under the influence of *V*. *apis* and *V*. *ceranae* infection. Other studies indicated that infection with *Vairimorpha* spp. caused a decrease in lysozyme expression [61].

The above literature data clearly indicate that the level of lysozyme in honeybee hemolymph is influenced by many factors, e.g. the presence of pathogens, age of the honeybees, state of the environment, and amount of protein in the food [62]. For comparison, bacterial (*Paenibacillus larvae*) and parasitic (*Varroa destructor*) infections also increase the level/activity of lysozyme in honeybees. Chan et al. [63] analyzed changes in the level of proteins, including lysozyme, in the hemolymph of 5-day-old larvae and reported that the level of lysozyme increased 13-fold under the influence of bacterial infection with *P*. *larvae*. Kunc et al. [64] found that the level of lysozyme in 10-day-old honeybees infected with *V*. *destructor* was twice as high as in the control, i.e. healthy honeybees after treatment against *Varroa* and healthy honeybees.

Interestingly, in our analyses of lysozyme-like activity, seasonality and the associated level of nosemosis infection proved to be important elements. In the study by Ptaszynska et al. [65], it was found that the level of infection, reflected by a number of *Vairimorpha* spp. spores, is highest in the spring months, then gradually decreases and reaches its lowest level in autumn. Similar observations have been reported recently by Kunat-Budzynska et al. [66]. Interestingly, our current study has shown that lysozyme-like activity was higher in 28-day-old honeybees during the summer months (June, July), when higher levels of infection were observed, compared to later months when the level of infection decreased, i.e. August and September (Fig 2), which may suggest an important role of lysozyme in the course of infection.

Another important immunological parameter is the PO activity. Literature data indicate a correlation between PO activity and insect`s resistance to parasitic, viral, bacterial, and fungal infections [67, 68]. Measurement of PO activity provides an opportunity to assess the condition of the honeybee colony, because an increase in PO activity is associated with increased levels of resistance to pathogens [69]. Together with the antioxidant system, the PO system participates in mitigating the effects of oxidative damage to the host cells and tissues [70].

The results of our research proved that the activity of PO is correlated with *Vairimorpha* infection, likewise the lysozyme-like activity. The highest PO activity was observed in all the months in honeybees living in their natural environment, i.e. in the apiary. In healthy and infected honeybees in June and July (summer months), PO activity increased with age, with the highest activity observed in 28-day-old honeybees. This is consistent with the observations of Schmid et al. [71] who found that PO activity increased with age in immunologically naïve workers and queens. However, in other studies by Spremo et al. [72], it was shown that in summer workers, the level of PO activity was independent their age. Other factors, such as environment or diet, had an impact on this activity. Under the influence of *V*. *ceranae* infection, we found an increase in the PO activity in most of the examined honeybees, compared to the control ‐ healthy honeybees (Fig 3). However, the differences in the PO activity between the healthy and infected honeybees in the laboratory in May and July were not so clear, but they were only noticeable in the later months, i.e. from July to September (Fig 3). Previous studies examined PO activity in response to nosemosis infection during the initial days of infection. In research conducted by Trytek et al. [40], the greatest increase in PO activity was recorded in honeybees on the third day after *V*. *ceranae* infection, while there was a decrease in PO activity on the subsequent infection day, compared to healthy honeybees. Similar conclusions were drawn in a study conducted by Antúnez et al. [54], where an increase in PO gene expression was observed on the first four days after *V*. *ceranae* infection and a decrease in PO gene expression was recorded after the seventh day of infection [54].

In the case of infestation by *V*. *destructor*, the PO activity in honeybees also increases. Millanta et al. [34] checked this parameter on days 0, 6, and 13 after emergence in three groups: control (honeybees naturally infected with this pathogen), superinfected (honeybees artificially infected with *V*. *destructor*), and one group administered with PBS. The greatest increase in the PO activity occurred on day 6 in the control group and on day 13 in the superinfected group, while the lowest activity was recorded in the PBS-treated group [34]. It is interesting that PO activity does not increase following bacterial infection with *Serratia marcescens*. This may be related to the fact that the strains of this bacterium were isolated from the gut of honeybees, which may indicate that this bacterium avoids the immune response of the host due to its pathogenicity to honeybees [73].

A very interesting study was carried out by Özgör [37], who compared the level of PO gene expression between the honeybee *A*. *mellifera* and the greater wax moth *Galleria mellonella*, i.e. a pest of apiaries. It was also checked whether *V*. *ceranae* and *V*. *apis* have the ability to colonize the gut of *G*. *mellonella*. The PO gene expression was examined in the gut of *G*. *mellonella* larvae in the second instar and 6, 9, and 12 days after infection of 1-day-old honeybees. In the case of *G*. *mellonella*, there was no increase in the level of phenoloxidase mRNA in any of the tested temporal variants in response to the *V*. *ceranae* or *V*. *apis* infection. It was also found that both of these pathogens can colonize the *G*. *mellonella* gut without causing the death of the host. This may indicate that *G*. *mellonella* is an asymptomatic vector of *V*. *ceranae* and *V*. *apis* in hives, which consequently leads to the spread of this disease in apiaries. In the honeybees, the highest level of phenoloxidase mRNA was observed on day 6 after the mixed infection. Moreover, the single infection increased the PO gene expression on days 9 and 12 of the experiment. An increase in PO mRNA levels may indicate an increase in protein levels, which can be manifested by an increase in the activity of this enzyme. This is in agreement with our results showing that infection with *V*. *ceranae* increases PO activity in honeybees. Nevertheless, phenoloxidase is stored in an inactive form called prophenoloxidase. As a result of infection caused by pathogens, the PO system is activated, which consequently leads to an increase in PO activity [33, 34]. Our research was conducted in two environments: artificial in the laboratory and in natural conditions (apiary). These two environments differ in various factors, i.e. in the laboratory, the honeybees were fed with sugar syrup, there was no queen honeybee, and the honeybees were not exposed to pesticides used by farmers, compared to the conditions in the apiary. The results of our experiments showed that the honeybees were exposed to the highest level of oxidative stress in the apiary. This situation may be related to the fact that oxidative stress is associated with flight. This relationship was confirmed in previous studies by Margotta et al. [14]. Flying honeybees showed higher levels of a marker associated with oxidative DNA damage (8-hydroxy-2′-deoxyguanosine) and hydrogen peroxide as well as reduced levels of antioxidants, e.g. catalase, compared to honeybees that had a lower possibility to fly. Additionally, other studies have demonstrated that oxidative stress increases with age and is high in foragers [13].

Another problem occurring in the apiary is a climate change. It may be responsible for heat stress, which in turn is also related to oxidative stress. Heat stress increases the production of ROS in honeybees and affects the growth and development of these insects [74]. Our results are in agreement with these observations, as the temperature in the laboratory was constant (25°C) throughout the entire experimental period, while the temperature in the apiary, where a higher total ROS/RNS level was detected, was high, especially in June (30°C) and August (31°C) (S2 Table in S1 File).

Nowadays, insecticides have increasingly been used in agriculture. Honeybees may be exposed to these insecticides through direct contact or by collecting contaminated pollen. The influence of small doses of pesticides (for example neonicotinoids or organophosphates) and infections caused by various pathogens lead to oxidative stress, which results in disruption of energy metabolism, inhibits the development, and accelerates aging of honeybees [48, 75]. Additionally, insecticides can cause increased susceptibility of honeybees to diseases, problems with orientation in the field and returning to the hive, and paralysis [4, 48, 75]. In our research, we did not check the use of pesticides, but we can assume that the honeybees may have been exposed to various types of pesticides, because the apiary is located in agricultural area. An example of a pesticide used in agriculture is the herbicide glyophosate, which is used to control weeds. Pons et al. [76] conducted laboratory experiments where honeybees were administered glyophosate and noted an increase in the expression of genes related to antioxidants and the activity of antioxidants, such as superoxide dismutase and catalase, proving that glyophosate causes oxidative stress in honeybees. *Vairimorpha ceranae* uses the honeybee’s midgut as an energy source for its replication. During this process, it damages honeybee’s epithelial cells, which leads to an increase in infection, thereby increasing the level of oxidative stress in honeybees [77]. In our experiments, the oxidative stress was higher mainly in the infected honeybees, compared to the healthy ones (Fig 4). Glavinic et al. [78] determined the activity of antioxidant enzymes, e.g. catalase, glutathione S-transferase, and superoxide dismutase, in homogenates from the whole healthy and *V*. *ceranae*-infected honeybees. The infected honeybees showed an increase in the activity of these enzymes, compared to the healthy honeybees [78]. Other studies [6] also reported an increase in the activity of antioxidant enzymes in the midgut, including superoxidase dismutase, indicating an increased level of ROS as an immune response to nosemosis infection, which is consistent with our observations. On the other hand, in the laboratory experiments Paris et al. [11] showed the opposite tendency, i.e. the amount of ROS was lower in the midguts of infected honeybees in most of the tested time variants than in the midguts of healthy honeybees.

In our study, the lysozyme-like activity, PO activity, and the level of oxidative stress were compared for the first time between healthy and *Vairimorpha-*infected honeybees kept in laboratory and natural (apiary) conditions. The available literature provides information that most experiments in which the above-mentioned parameters were determined were conducted in laboratory conditions [6, 15, 35, 37, 40, 45, 73, 75]. Our previous studies have shown that, among others, keeping conditions had a significant impact on the occurrence of pathogens and the level of their infection in honeybees. One such example is the finding of trypanosomatids in honeybees kept in an apiary, but not in laboratory-reared honeybees. The opposite trend can be seen in the case of infection with Black Queen Cell Virus, which occurred more often in the laboratory compared to the apiary (Kunat-Budzyńska et al. 2024). There is a great need for such comparative studies, as they allow us to understand to what extent the results of laboratory tests reflect the actual conditions that exist in honeybee colonies in apiaries. We have evidenced that natural/laboratory conditions have a significant impact on the tested parameters. The results of our research indicate that in the natural apiary conditions there is a higher level of oxidative stress and lysozyme-like and PO activity, compared to artificial conditions (laboratory) (Figs 2–4).

## 5. Conclusions

Immune mechanisms in honeybees remain largely unexplored due to the complex social structure of these insects. Our study clearly shows that keeping conditions (apiary vs. laboratory) are extremely important and have a very strong impact on oxidative stress level, phenoloxidase and lysozyme activity, as honeybees kept in the apiary had increased these immune parameters. Therefore, the results of research conducted in the laboratory will not always reflect the data obtained in the apiary, as clearly presented for the first time in this paper. Also, nosemosis infection is a challenge for the immune and antioxidative systems. The pathogen *Vairimorpha* spp. significantly affects insect ‐ homeostasis and requires a rapid response of honeybee defense systems. This response is characterized by an increase in the activity of the immune and antioxidative mechanisms, which indicates the mobilization of forces to fight the infection and minimize its effects. Seasonality also proved to be an important factor in our studies. Honeybees show seasonal differences in lysozyme-like activity, however no such changes were detected in the level of phenoloxidase activity and oxidative stress. It can be postulated that different defense mechanisms are involved to varying degrees in response to changing seasonal conditions, with some of them being influenced and others being: independent of seasonal environmental changes.

## Supporting information

S1 File Tables, figures and data. (DOCX)

S2 File Statistical summaries. (XLSX)

S1 Fig 2% agarose gel showing *V*. *ceranae* PCR products (219 bp) in honeybees collected in May. The designations of the samples of honeybees are shown in S1 Table. M–DNA Ladder (100 bp).
(TIF)

S2 Fig 2% agarose gel showing *V*. *ceranae* PCR products (219 bp) in honeybees collected in June. The designations of the samples of honeybees are shown in S1 Table. M–DNA Ladder (100 bp).
(TIF)

S3 Fig 2% agarose gel showing *V*. *ceranae* PCR products (219 bp) in honeybees collected in July. The designations of the samples of honeybees are shown in S1 Table. M–DNA Ladder (100 bp).
(TIF)

S4 Fig 2% agarose gel showing *V*. *ceranae* PCR products (219 bp) in honeybees collected in August. The designations of the samples of honeybees are shown in S1 Table. M–DNA Ladder (100 bp).
(TIF)

S5 Fig 2% agarose gel showing *V*. *ceranae* PCR products (219 bp) in honeybees collected in September. The designations of the samples of honeybees are shown in S1 Table. M–DNA Ladder (100 bp).; Raw data, Originals gels.
(TIF)

## References

[1] Girotti S, Ghini S, Ferri E, Bolelli L, Colombo R, Serra G, et al. Bioindicators and biomonitoring: honeybees and hive products as pollution impact assessment tools for the Mediterranean area. Euro-Mediterr. J. Environ. Integr. 2020;5:62. doi: 10.1007/s41207-020-00204-9

[2] La Porta G, Magara G, Goretti E, Caldaroni B, Dörr AJM, Selvaggi R, et al. Applying artificial neural networks to oxidative stress biomarkers in forager honey bees (*Apis mellifera*) for ecological assessment. Toxics. 2023;11(8):661. doi: 10.3390/toxics11080661 37624166 PMC10459414

[3] Li G, Zhao H, Liu Z, Wang H, Xu B, Guo X. The wisdom of honeybee defenses against environmental stresses. Front Microbiol. 2018;9:722. doi: 10.3389/fmicb.2018.00722 29765357 PMC5938604

[4] Olgun T, Dayıoğlu M, Taskiran N. Pesticide and pathogen induced oxidative stress in honey bees (*Apis mellifera* L.). Mellifera. 2020;20;32–52.

[5] Lin Z, Shen S, Wang K, Ji T. Biotic and abiotic stresses on honeybee health. Integr Zool. 2024;19(3):442–457. doi: 10.1111/1749-4877.12752 37427560

[6] Dussaubat C, Brunet JL, Higes M, Colbourne JK, Lopez J, Choi JH, et al. Gut pathology and responses to the microsporidium *Nosema ceranae* in the honey bee *Apis mellifera*. PLoS One. 2012;7(5):e37017. doi: 10.1371/journal.pone.0037017 22623972 PMC3356400

[7] Jovanovic NM, Glavinic U, Ristanic M, Vejnovic B, Ilic T, Stevanovic J, et al. Effects of plant-based supplement on oxidative stress of honey bees (*Apis mellifera*) infected with *Nosema ceranae*. Animals (Basel). 2023;13(22):3543. doi: 10.3390/ani13223543 38003159 PMC10668651

[8] Tahir F, Goblirsch M, Adamczyk, Karim S, Alburaki M. Honey bee *Apis mellifera* L. responses to oxidative stress induced by pharmacological and pesticide compounds. Front Bee Sci. 2023;1:127586210. doi: 10.3389/frbee.2023.1275862

[9] Simone-Finstrom M, Li-Byarlay H, Huang M, Strand MK, Rueppell O, Tarpy DR. Migratory management and environmental conditions affect lifespan and oxidative stress in honey bees. Sci Rep. 2016;6:32023. doi: 10.1038/srep32023 27554200 PMC4995521

[10] Słowińska M, Nynca J, Wilde J, Bąk B, Siuda M, Ciereszko A. Total antioxidant capacity of honeybee haemolymph in relation to age and exposure to pesticide, and comparison to antioxidant capacity of seminal plasma. Apidologie. 2016;47:227–236. doi: 10.1007/s13592-015-0391-9

[11] Paris L, Roussel M, Pereira B, Delbac F, Diogon M. Disruption of oxidative balance in the gut of the western honeybee *Apis mellifera* exposed to the intracellular parasite *Nosema ceranae* and to the insecticide fipronil. Microb Biotechnol. 2017;10(6):1702–1717. doi: 10.1111/1751-7915.12772 28736933 PMC5658624

[12] Ward K, Cleare X, Li-Byarlay H. The life span and levels of oxidative stress in foragers between feral and managed honey bee colonies. J Insect Sci. 2022;22(1):20. doi: 10.1093/jisesa/ieac002 35137132 PMC8826185

[13] Vance JT, Williams JB, Elekonich MM, Roberts SP. The effects of age and behavioral development on honey bee (*Apis mellifera*) flight performance. J Exp Biol. 2009;212:2604–11. doi: 10.1242/jeb.028100 19648405 PMC2726856

[14] Margotta JW, Roberts SP, Elekonich MM. Effects of flight activity and age on oxidative damage in the honey bee, *Apis mellifera*. J Exp Biol. 2018;221:jeb183228. doi: 10.1242/jeb.183228 29724776

[15] Kunat M, Wagner GK, Staniec B, Jaszek M, Matuszewska A, Stefaniuk D, et al. Aqueous extracts of jet-black ant *Lasius fuliginosus* nests for controlling nosemosis, a disease of honeybees caused by fungi of the genus *Nosema*. Eur Zool J. 2020;87:770–780. doi: 10.1080/24750263.2020.1845405

[16] Marín-García PJ, Peyre Y, Ahuir-Baraja AE, Garijo MM, Llobat L. The role of *Nosema ceranae* (Microsporidia: Nosematidae) in honey bee colony losses and current insights on treatment. Vet Sci. 2022;9(3):130. doi: 10.3390/vetsci9030130 35324858 PMC8952814

[17] García‐Palencia P, Martín‐Hernández R, Marin P, Meana A, Higes M. Natural infection by *Nosema ceranae* causes similar lesions as in experimentally infected caged‐worker honey bees (*Apis mellifera*). J Apic Res. 2010;49:278–283. doi: 10.3896/IBRA.1.49.3.08

[18] Ptaszyńska AA, Gancarz M. Microsporidiosis causing necrotic changes in the honeybee intestine. Appl. Sci. 2023;13:4957. doi: 10.3390/app13084957

[19] Jack CJ, Uppala SS, Lucas HM, Sagili RR. Effects of pollen dilution on infection of *Nosema ceranae* in honey bees. J Insect Physiol. 2016;87:12–19. doi: 10.1016/j.jinsphys.2016.01.004 26802559

[20] Vidau C, Panek J, Texier C, Biron DG, Belzunces LP, Le Gall M, et al. Differential proteomic analysis of midguts from *Nosema ceranae*-infected honeybees reveals manipulation of key host functions. J Invertebr Pathol. 2014;121:89–96. doi: 10.1016/j.jip.2014.07.002 25038465

[21] Huang Q, Wu ZH, Li WF, Guo R, Xu JS, Dang XQ, et al. Genome and evolutionary analysis of *Nosema ceranae*: A microsporidian parasite of honey bees. Front Microbiol. 2021;2:12:645353. doi: 10.3389/fmicb.2021.645353 34149635 PMC8206274

[22] Dussaubat C, Maisonnasse A, Alaux C, Tchamitchan S, Brunet JL, Plettner E, et al. Nosema spp. infection alters pheromone production in honey bees (*Apis mellifera*). J Chem Ecol. 2010;36(5):522–5. doi: 10.1007/s10886-010-9786-2 20401523

[23] Barroso-Arévalo S, Vicente-Rubiano M, Puerta F, Molero F, Sánchez-Vizcaíno JM. Immune related genes as markers for monitoring health status of honey bee colonies. BMC Vet Res. 2019;15(1):72. doi: 10.1186/s12917-019-1823-y 30832657 PMC6398266

[24] Morfin N, Anguiano-Baez R, Guzman-Novoa E. Honey Bee (*Apis mellifera*) Immunity. Vet Clin North Am Food Anim Pract. 2021;37(3):521–533. doi: 10.1016/j.cvfa.2021.06.007 34689918

[25] Evans JD, Aronstein K, Chen YP, Hetru C, Imler JL, Jiang H, et al. Immune pathways and defence mechanisms in honey bees *Apis mellifera*. Insect Mol Biol. 2006;15(5):645–56. doi: 10.1111/j.1365-2583.2006.00682.x 17069638 PMC1847501

[26] Al-Ghamdi AA, Al-Ghamdi MS, Ahmed AM, Mohamed ASA, Shaker GH, Ansari MJ, et al. Immune investigation of the honeybee *Apis mellifera jemenitica* broods: A step toward production of a bee-derived antibiotic against the American foulbrood. Saudi J Biol Sci. 2021;28(3):1528–1538. doi: 10.1016/j.sjbs.2020.12.026 33732036 PMC7938142

[27] Beckert A, Wiesner J, Schmidtberg H, Lehmann R, Baumann A, Vogel H, et al. Expression and characterization of a recombinant i-type lysozyme from the harlequin ladybird beetle *Harmonia axyridis*. Insect Mol Biol. 2016;25(3):202–15. doi: 10.1111/imb.12213 26778648

[28] Zaobidna E, Zoltowska K, Łopieńska-Biernat E. Expression and activity of lysozyme in *Apis mellifera* carnica brood infested with varroa destructor. J Apic Sci. 2017;61. doi: 10.1515/jas-2017-0014

[29] Chapelle M, Girard PA, Cousserans F, Volkoff NA, Duvic B. Lysozymes and lysozyme-like proteins from the fall armyworm, Spodoptera frugiperda. Mol Immunol. 2009;47(2–3):261–9. doi: 10.1016/j.molimm.2009.09.028 19828200

[30] Doublet V, Poeschl Y, Gogol-Döring A, Alaux C, Annoscia D, Aurori C, et al. Unity in defence: honeybee workers exhibit conserved molecular responses to diverse pathogens. BMC Genomics. 2017;18:207. doi: 10.1186/s12864-017-3597-6 28249569 PMC5333379

[31] Luz GFD, Santana WC, Santos CG, Medeiros Santana L, Serrão JE. Cuticle melanization and the expression of immune-related genes in the honeybee *Apis mellifera* (Hymenoptera: Apidae) adult workers. Comp Biochem Physiol B Biochem Mol Biol. 2022;257:110679. doi: 10.1016/j.cbpb.2021.110679 34673246

[32] Andrejko M. Modulation of the humoral immune response in *Galleria mellonella* larvae by proteolytic enzymes produced by *Pseudomonas aeruginosa*. Postępy Mikrobiol. 2016;55:255–267 (Paper in Polish).

[33] Stączek S, Grygorczuk K, Zdybicka-Barabas A, Siemińska-Kuczer A, Vertyporokh L, Andrejko M, et al. Different faces of phenoloxidase in animals, Postępy Biochem. 2017;63:315–325 (Paper in Polish). 29374432

[34] Millanta F, Sagona S, Mazzei M, Forzan M, Poli A, Felicioli A. Phenoloxidase activity and haemolymph cytology in honeybees challenged with a virus suspension (*deformed wings virus* DWV) or phosphate buffered suspension (PBS). Cienc Rural. 2019;49:e20180726. doi: 10.1590/0103-8478cr20180726

[35] Alaux C, Brunet JL, Dussaubat C, Mondet F, Tchamitchan S, Cousin M, et al. Interactions between *Nosema* microspores and a neonicotinoid weaken honeybees (*Apis mellifera*). Environ Microbiol. 2010;12(3):774–82. doi: 10.1111/j.1462-2920.2009.02123.x 20050872 PMC2847190

[36] Felicioli A, Forzan M, Sagona S, D’Agostino P, Baido D, Fronte B, et al. Effect of oral administration of 1,3–1,6 β-Glucans in DWV naturally infected newly emerged bees (*Apis mellifera* L.). Vet Sci. 2020;7(2):52. doi: 10.3390/vetsci7020052 32344871 PMC7355867

[37] Özgör E. The Effects of Nosema apis and *Nosema ceranae* Infection on Survival and Phenoloxidase Gene Expression in *Galleria mellonella* (Lepidoptera: Galleriidae) Compared to *Apis mellifera*. Insects. 2021; 12: 953. doi: 10.3390/insects12100953 34680722 PMC8538655

[38] Ptaszyńska AA, Trytek M, Borsuk G, Buczek K, Rybicka-Jasińska K, Gryko D. Porphyrins inactivate *Nosema* spp. microsporidia. Sci Rep. 2018;8(1):5523. doi: 10.1038/s41598-018-23678-8 29615690 PMC5882804

[39] Sulborska A., Horecka B., Cebrat M. et al. Microsporidia *Nosema* spp.–obligate bee parasites are transmitted by air. Sci Rep. 2019;9:14376. doi: 10.1038/s41598-019-50974-8 31591451 PMC6779873

[40] Trytek M, Buczek K, Zdybicka-Barabas A, Wojda I, Borsuk G, Cytryńska M, et al. Effect of amide protoporphyrin derivatives on immune response in *Apis mellifera*. Sci Rep. 2022;12(1):14406. doi: 10.1038/s41598-022-18534-9 36002552 PMC9402574

[41] Martín-Hernández R, Meana A, García-Palencia P, Marín P, Botías C, Garrido-Bailón E, et al. Effect of temperature on the biotic potential of honeybee microsporidia. Appl Environ Microbiol. 2009;75(8):2554–7. doi: 10.1128/AEM.02908-08 19233948 PMC2675226

[42] Forsgren E, Fries I. Comparative virulence of *Nosema ceranae* and *Nosema apis* in individual European honey bees. Vet Parasitol. 2010;170:212–217. doi: 10.1016/j.vetpar.2010.02.010 20299152

[43] Fries I, Chauzat MP, Chen YP, Doublet V, Genersch E, Gisder S, et al. Standard methods for *Nosema* research. J Apic Res. 2013;51:1–28.

[44] Borsuk G., Ptaszyńska A.A., Olszewski K., Domaciuk M., Krutmuang P., Paleolog J. 2017. A new method for quick and easy hemolymph collection from Apidae adults. PLoS ONE 12(1): e0170487. doi: 10.1371/journal.pone.0170487 28125668 PMC5268409

[45] Ptaszyńska AA, Borsuk G, Zdybicka-Barabas A, Cytryńska M, Małek W. Are commercial probiotics and prebiotics effective in the treatment and prevention of honeybee nosemosis C? Parasitol Res. 2016;115(1):397–406. doi: 10.1007/s00436-015-4761-z 26437644 PMC4700093

[46] Andrejko M, Mizerska-Dudka M, Jakubowicz T. Changes in *Galleria mellonella* lysozyme level and activity during *Pseudomonas aeruginosa* infection. Folia Microbiol. 2008;53:147–151. doi: 10.1007/s12223-008-0021-2 18500634

[47] Ptaszyńska AA, Borsuk G, Mułenko WJ. Wilk J. Impact of vertebrate probiotics on honeybee yeast microbiota and on the course of nosemosis, Med Weter. 2016;72:430–434.

[48] Chakrabarti P, Carlson EA, Lucas HM, Melathopoulos AP, Sagili RR. Field rates of Sivanto^™^ (flupyradifurone) and Transform^®^ (sulfoxaflor) increase oxidative stress and induce apoptosis in honey bees (*Apis mellifera* L.). PLOS ONE. 2020;15:e0233033. doi: 10.1371/journal.pone.0233033 32437365 PMC7241780

[49] Copley TR, Jabaji SH. Honeybee glands as possible infection reservoirs of *Nosema ceranae* and *Nosema apis* in naturally infected forager bees. J Appl Microbiol. 2012;112(1):15–24. doi: 10.1111/j.1365-2672.2011.05192.x 22053729

[50] Ansari MJ, Al-Ghamdi A, Nuru A, Khan KA, Alattal Y. Geographical distribution and molecular detection of *Nosema ceranae* from indigenous honey bees of Saudi Arabia. Saudi J Biol Sci. 2017;24(5):983–991. doi: 10.1016/j.sjbs.2017.01.054 28663692 PMC5478367

[51] Branchiccela B, Arredondo D, Higes M, Invernizzi C, Martín-Hernández R, Tomasco I, et al. Characterization of *Nosema ceranae* Genetic Variants from Different Geographic Origins. Microb Ecol. 2017;73(4):978–987. doi: 10.1007/s00248-016-0880-z 27837253

[52] Yazlovytska LS, Karavan VV, Domaciuk M, Panchuk II, Borsuk G, Volkov RA. Increased survival of honey bees consuming pollen and beebread is associated with elevated biomarkers of oxidative stress. Front Ecol Evol. 2023;11:1098350. doi: 10.3389/fevo.2023.1098350

[53] Imler JL, Bulet P. Antimicrobial peptides in Drosophila: structures, activities and gene regulation. Chem Immunol Allergy. 2005;86:1–21. doi: 10.1159/000086648 15976485

[54] Antúnez K, Martín-Hernández R, Prieto L, Meana A, Zunino P, Higes M. Immune suppression in the honey bee (*Apis mellifera*) following infection by *Nosema ceranae* (Microsporidia). Environ Microbiol. 2009;11(9):2284–90. doi: 10.1111/j.1462-2920.2009.01953.x 19737304

[55] Castelli L, Branchiccela B, Garrido M, Invernizzi C, Porrini M, Romero H, et al. Impact of nutritional stress on honeybee gut microbiota, immunity, and *Nosema ceranae* Infection. Microb Ecol. 2020;80(4):908–919. doi: 10.1007/s00248-020-01538-1 32666305

[56] Leitch GJ, Ceballos C. A role for antimicrobial peptides in intestinal microsporidiosis. Parasitology. 2009;136(2):175–81. doi: 10.1017/S0031182008005313 19079820 PMC2743560

[57] Sinpoo C, Paxton RJ, Disayathanoowat T, Krongdang S, Chantawannakul P. Impact of *Nosema ceranae* and *Nosema apis* on individual worker bees of the two host species (*Apis cerana* and *Apis mellifera*) and regulation of host immune response. J Insect Physiol. 2018;105:1–8. doi: 10.1016/j.jinsphys.2017.12.010 29289505

[58] Sowa-Jasiłek A., Zdybicka-Barabas A., Stączek S., Wydrych J., Skrzypiec K., Mak P., et al., *Galleria mellonella* lysozyme induces apoptopic changes in *Candida albicans* cells, Microbiol Res. 2016;193:121–131. doi: 10.1016/j.micres.2016.10.003 27825480

[59] Callewaert L, Michiels CW, Lysozymes in the animal kingdom. J Biosci. 2010;35:127–160. doi: 10.1007/s12038-010-0015-5 20413917

[60] Ma Z, Wang Y, Huang Z, Cheng S, Xu J, Zhou Z. Isolation of protein-free chitin spore coats of *Nosema ceranae* and its application to screen the interactive spore wall proteins. Arch Microbiol. 2021;203(5):2727–2733. doi: 10.1007/s00203-021-02214-9 33646339

[61] Garrido PM, Porrini MP, Antúnez K, Branchiccela B, Martínez-Noël GM, Zunino P, et al. Sublethal effects of acaricides and *Nosema ceranae* infection on immune related gene expression in honeybees. Vet Res. 2016;47(1):51. doi: 10.1186/s13567-016-0335-z 27118545 PMC4847213

[62] Lazarov S, Zhelyazkova I, Salkova D, Shumkova R, Takova S. Lysozyme levels in haemolymph of worker bees (*Apis mellifera* L.) from bee colonies with different degree of expression of hygienic behaviour. Agric Sci Technol. 2016;8:201–204. doi: 10.15547/ast.2016.03.037

[63] Chan QW, Melathopoulos AP, Pernal SF, Foster LJ. The innate immune and systemic response in honey bees to a bacterial pathogen, *Paenibacillus larvae*. BMC Genomics. 2009;10:387. doi: 10.1186/1471-2164-10-387 19695106 PMC2907699

[64] Kunc M, Dobeš P, Ward R, Lee S, Čegan R, Dostálková S, et al. Omics-based analysis of honey bee (*Apis mellifera*) response to *Varroa* sp. parasitisation and associated factors reveals changes impairing winter bee generation. Insect Biochem Mol Biol. 2023;152:103877. doi: 10.1016/j.ibmb.2022.103877 36403678

[65] Ptaszyńska AA, Paleolog J, Borsuk G (2016) *Nosema ceranae* Infection Promotes Proliferation of Yeasts in Honey Bee Intestines. PLoS ONE 11(10): e0164477. doi: 10.1371/journal.pone.0164477 27736915 PMC5063367

[66] Kunat-Budzyńska M, Łabuć E, Ptaszyńska AA. Seasonal detection of pathogens in honeybees kept in natural and laboratory conditions. Parasitol Int. 2024;104:102978. doi: 10.1016/j.parint.2024.102978 39378965

[67] Wilson-Rich N, Dres ST, Starks PT. The ontogeny of immunity: development of innate immune strength in the honey bee (*Apis mellifera*). J Insect Physiol. 2008;54(10–11):1392–9. doi: 10.1016/j.jinsphys.2008.07.016 18761014

[68] Santoyo I, Córdoba-Aguilar A. Phenoloxidase: A key component of the insect immune system. Entomol Exp Appl. 2012;142:1–16. doi: 10.1111/j.1570-7458.2011.01187.x

[69] Laughton AM, Boots M, Siva-Jothy MT. The ontogeny of immunity in the honey bee, *Apis mellifera* L. following an immune challenge. J Insect Physiol. 2011;57(7):1023–32. doi: 10.1016/j.jinsphys.2011.04.020 21570403

[70] Saltykova E.S., Gaifullina L.R., Kaskinova M.D. et al. Effect of Chitosan on Development of *Nosema apis* microsporidia in honey bees. Microbiolog*y*. 2018;87:738–743. doi: 10.1134/S0026261718050144

[71] Schmid MR, Brockmann A, Pirk CW, Stanley D.W, Tautz J. Adult honeybees (Apis mellifera L.) abandon hemocytic, but not phenoloxidase-based immunity. J Insect Physiol. 2008;54:439–444. doi: 10.1016/j.jinsphys.2007.11.002 18164310

[72] Spremo J, Purać J, Čelić T, Đorđievski S, Pihler I, Kojić D, et al. Assessment of oxidative status, detoxification capacity and immune responsiveness in honey bees with ageing. Comp Biochem Physiol A Mol Integr Physiol. 2024;298:111735. doi: 10.1016/j.cbpa.2024.111735 39233113

[73] Raymann K, Coon K, Shaffer Z, Salisbury S, Moran N. Pathogenicity of *Serratia marcescens* strains in honey bees. mBio. 2018;9. doi: 10.1128/mBio.01649-18 30301854 PMC6178626

[74] Zhao H, Li G, Guo D, Li H, Liu Q, Baohua X, et al. Response mechanisms to heat stress in bees. Apidologie. 2021;52. doi: 10.1007/s13592-020-00830-w

[75] Liu L, Shi M, Wu Y, Xie X, Li S, Dai P, et al. Interactive effects of dinotefuran and *Nosema ceranae* on the survival status and gut microbial community of honey bees. Pestic Biochem Physiol. 2024;200:105808. doi: 10.1016/j.pestbp.2024.105808 38582580

[76] Pons DG, Herrera C, Torrens-Mas M, Leza M, Sastre-Serra J. Sublethal doses of glyphosate modulates mitochondria and oxidative stress in honeybees by direct feeding. Arch Insect Biochem Physiol. 2023;114(1):e22028. doi: 10.1002/arch.22028 37259187

[77] Gage SL, Kramer C, Calle S, Carroll M, Heien M, DeGrandi-Hoffman G. *Nosema ceranae* parasitism impacts olfactory learning and memory and neurochemistry in honey bees (*Apis mellifera*). J Exp Biol. 2018;221(Pt 4):jeb161489. doi: 10.1242/jeb.161489 29361577

[78] Glavinic U, Dzogovic D, Jelisić S, Ristanić M, Zorc M, Aleksic N, et al. Oxidative status of honey bees infected with *Nosema ceranae* microsporidium and supplemented with *Agaricus bisporus* mushroom extract. Veterinarski Glasnik. 2022;77:13–13. doi: 10.2298/VETGL220715013G

