# Supplementary material for: Changes in enzymatic activity and oxidative stress in honeybees kept in the apiary and laboratory conditions during the course of nosemosis
Source: PLoS One. 2025 Jan 15;20(1):e0317384. doi: 10.1371/journal.pone.0317384 (PMC11734893; doi:10.1371/journal.pone.0317384)
Supplement: S1 File — (DOCX) [file pone.0317384.s001.docx]

**S1 Table****. The designations of honeybee variants at nucleic acid electrophoresis.**

| **Number samples** | **Honeybee variants** |
| --- | --- |
| 1, 2, 3 | 1-day-old honeybees from group 1 (three biological repeats) |
| 4, 5, 6 | 10-day-old honeybees from group 1L (three biological repeats) |
| 7, 8, 9 | 10-day-old honeybees from group 1A (three biological repeats) |
| 10, 11, 12 | 19-day-old honeybees from group 1L (three biological repeats) |
| 13, 14, 15 | 19-day-old honeybees from group 1A (three biological repeats) |
| 16, 17, 18 | 28-day-old honeybees from group 1L (three biological repeats) |
| 19, 20, 21 | 28-day-old honeybees from group 1A (three biological repeats) |
| 22, 23, 24 | 1-day-old honeybees from group 2 (three biological repeats) |
| 25, 26, 27 | 10-day-old honeybees from group 2L (three biological repeats) |
| 28, 29, 30 | 10-day-old honeybees from group 2A (three biological repeats) |
| 31, 32, 33 | 19-day-old honeybees from group 2L (three biological repeats) |
| 34, 35, 36 | 19-day-old honeybees from group 2A (three biological repeats) |
| 37, 38, 39 | 28-day-old honeybees from group 2L (three biological repeats) |
| 40, 41, 42 | 28-day-old honeybees from group 2A (three biological repeats) |

**S2 Table. Temperature conditions in the apiary during the collection of selected honeybee variants for experiments.**

|  | **Age of worker honeybees** | **Month** | **Average temperature** |
| --- | --- | --- | --- |
| Group 1A and 2A | 19-day-old | May | 15ºC |
|  |  | June | 28ºC |
|  |  | July | 27ºC |
|  |  | August | 24ºC |
|  |  | September | 24ºC |
|  | 28-day-old | May | 21ºC |
|  |  | June | 30ºC |
|  |  | July | 28ºC |
|  |  | August | 31ºC |
|  |  | September | 15ºC |





**S1 Fig.** **2% agarose gel showing *V. ceranae* PCR products in honeybees collected in May. The designations of the samples of honeybees are shown in Table S1. M – DNA Ladder (100 bp).**


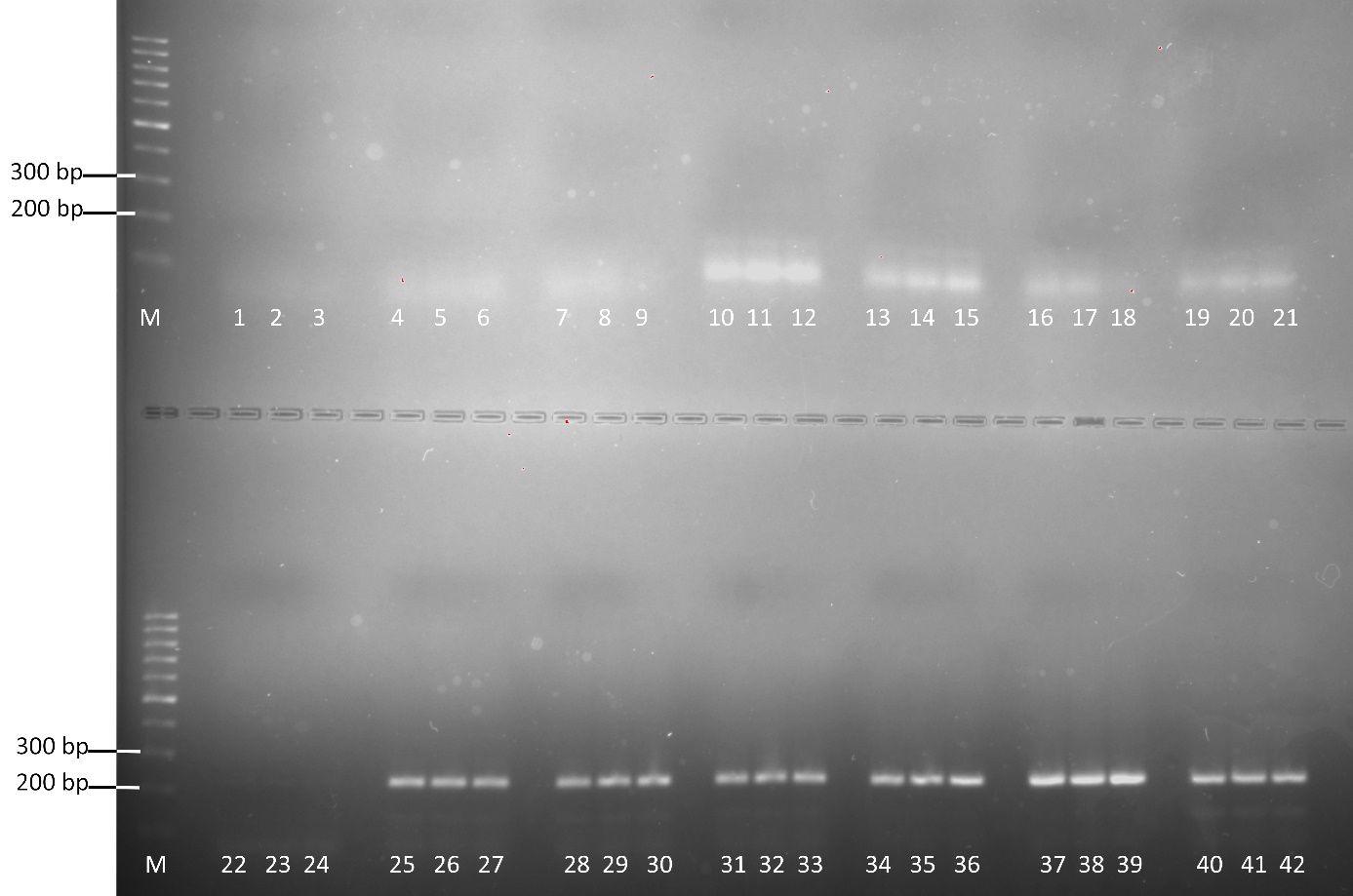


**S2 Fig.** **2% agarose gel showing *V. ceranae* PCR products in honeybees collected in June. The designations of the samples of honeybees are shown in Table S1. M – DNA Ladder (100 bp).**





**S3 Fig.** **2% agarose gel showing *V. ceranae* PCR products in honeybees collected in July. The designations of the samples of honeybees are shown in Table S1. M – DNA Ladder (100 bp).**





**S4 Fig.** **2% agarose gel showing *V. ceranae* PCR products in honeybees collected in August. The designations of the samples of honeybees are shown in Table S1. M – DNA Ladder (100 bp).**





**S5 Fig. 2% agarose gel showing *V. ceranae* PCR products in honeybees collected in September. The designations of the samples of honeybees are shown in Table S1. M – DNA Ladder (100 bp).**

**Raw data**

Lysozyme-like activity, May

| Healthy | I REPETITION | II REPETITION | III REPETITION |
| --- | --- | --- | --- |
| 1-day-old | 0.81 | 0.85 | 0.55 |
| 19-day-old lab | 0.31 | 0.29 | 0.32 |
| 19-day-old apiary | 0.36 | 0.34 | 0.38 |
| 28-day-old lab | 1.44 | 1 | 1.34 |
| 28-day-old apiary | 5.07 | 6.45 | 5.71 |
|  |  |  |  |
| Infected | I REPETITION | II REPETITION | III REPETITION |
| 1-day-old | 0.57 | 0.53 | 0.52 |
| 19-day-old lab | 0.35 | 0.37 | 0.32 |
| 19-day-old apiary | 0.42 | 0.47 | 0.5 |
| 28-day-old lab | 1.62 | 1.7 | 1.82 |
| 28-day-old apiary | 1.4 | 1.67 | 1.37 |

Lysozyme-like activity, June

| Healthy | I REPETITION | II REPETITION | III REPETITION |
| --- | --- | --- | --- |
| 1-day-old | 0.43 | 0.37 | 0.55 |
| 19-day-old lab | 0.26 | 0.32 | 0.29 |
| 19-day-old apiary | 0.65 | 0.71 | 0.63 |
| 28-day-old lab | 1.01 | 1.28 | 1.21 |
| 28-day-old apiary | 3.41 | 4.19 | 3.96 |
|  |  |  |  |
| Infected | I REPETITION | II REPETITION | III REPETITION |
| 1-day-old | 0.34 | 0.26 | 0.28 |
| 19-day-old lab | 0.53 | 0.42 | 0.45 |
| 19-day-old apiary | 3.46 | 3.32 | 3.03 |
| 28-day-old lab | 1.98 | 1.84 | 2.38 |
| 28-day-old apiary | 11.73 | 13.97 | 12.35 |

Lysozyme-like activity, July

| Healthy | I REPETITION | II REPETITION | III REPETITION |
| --- | --- | --- | --- |
| 1-day-old | 0.46 | 0.5 | 0.54 |
| 19-day-old lab | 0.57 | 0.5 | 0.52 |
| 19-day-old apiary | 1.63 | 1.73 | 1.47 |
| 28-day-old lab | 1.68 | 1.58 | 1.63 |
| 28-day-old apiary | 4.79 | 4.41 | 4.23 |
|  |  |  |  |
| Infected | I REPETITION | II REPETITION | III REPETITION |
| 1-day-old | 0.54 | 0.51 | 0.58 |
| 19-day-old lab | 0.67 | 0.63 | 0.75 |
| 19-day-old apiary | 4.89 | 4.57 | 5.12 |
| 28-day-old lab | 3.1 | 2.86 | 3.43 |
| 28-day-old apiary | 6.97 | 7.85 | 7.27 |

Lysozyme-like activity, August

| Healthy | I REPETITION | II REPETITION | III REPETITION |
| --- | --- | --- | --- |
| 1-day-old | 0.28 | 0.30 | 0.33 |
| 19-day-old lab | 0.31 | 0.28 | 0.32 |
| 19-day-old apiary | 0.49 | 0.53 | 0.55 |
| 28-day-old lab | 0.32 | 0.34 | 0.36 |
| 28-day-old apiary | 0.40 | 0.38 | 0.42 |
|  |  |  |  |
| Infected | I REPETITION | II REPETITION | III REPETITION |
| 1-day-old | 0.35 | 0.27 | 0.30 |
| 19-day-old lab | 0.36 | 0.41 | 0.44 |
| 19-day-old apiary | 0.45 | 0.44 | 0.42 |
| 28-day-old lab | 1.02 | 1.06 | 1.09 |
| 28-day-old apiary | 1.26 | 1.53 | 1.69 |

Lysozyme-like activity, September

| Healthy | I REPETITION | II REPETITION | III REPETITION |
| --- | --- | --- | --- |
| 1-day-old | 0.39 | 0.43 | 0.38 |
| 19-day-old lab | 1.26 | 1.13 | 1.02 |
| 19-day-old apiary | 1.69 | 1.58 | 1.80 |
| 28-day-old lab | 1.07 | 1.16 | 1.02 |
| 28-day-old apiary | 2.64 | 1.67 | 1.50 |
|  |  |  |  |
| Infected | I REPETITION | II REPETITION | III REPETITION |
| 1-day-old | 0.46 | 0.53 | 0.50 |
| 19-day-old lab | 1.58 | 1.47 | 1.84 |
| 19-day-old apiary | 2.48 | 2.10 | 2.43 |
| 28-day-old lab | 0.98 | 1.20 | 1.13 |
| 28-day-old apiary | 3.09 | 4.06 | 3.74 |

Phenoloxidase activity, May

| Healthy | I REPETITION | II REPETITION | III REPETITION |
| --- | --- | --- | --- |
| 1-day-old | 0.09 | 0.09 | 0.09 |
| 19-day-old lab | 0.106 | 0.084 | 0.086 |
| 19-day-old apiary | 0.09 | 0.09 | 0.088 |
| 28-day-old lab | 0.086 | 0.084 | 0.086 |
| 28-day-old apiary | 0.518 | 0.408 | 0.692 |
|  |  |  |  |
| Infected | I REPETITION | II REPETITION | III REPETITION |
| 1-day-old | 0.086 | 0.092 | 0.094 |
| 19-day-old lab | 0.09 | 0.084 | 0.096 |
| 19-day-old apiary | 0.158 | 0.142 | 0.154 |
| 28-day-old lab | 0.076 | 0.09 | 0.164 |
| 28-day-old apiary | 3.504 | 3.55 | 3.616 |

Phenoloxidase activity, June

| Healthy | I REPETITION | II REPETITION | III REPETITION |
| --- | --- | --- | --- |
| 1-day-old | 0.094 | 0.094 | 0.094 |
| 19-day-old lab | 0.144 | 0.182 | 0.146 |
| 19-day-old apiary | 0.514 | 0.708 | 0.622 |
| 28-day-old lab | 0.124 | 0.152 | 0.138 |
| 28-day-old apiary | 0.658 | 0.632 | 0.36 |
|  |  |  |  |
| Infected | I REPETITION | II REPETITION | III REPETITION |
| 1-day-old | 0.128 | 0.122 | 0.096 |
| 19-day-old lab | 0.182 | 0.162 | 0.174 |
| 19-day-old apiary | 3.318 | 3.486 | 3.376 |
| 28-day-old lab | 0.316 | 0.216 | 0.256 |
| 28-day-old apiary | 2.926 | 3.02 | 2.962 |

Phenoloxidase activity, July

| Healthy | I REPETITION | II REPETITION | III REPETITION |
| --- | --- | --- | --- |
| 1-day-old | 0.11 | 0.11 | 0.10 |
| 19-day-old lab | 0.14 | 0.16 | 0.14 |
| 19-day-old apiary | 3.69 | 3.83 | 3.40 |
| 28-day-old lab | 0.23 | 0.20 | 0.37 |
| 28-day-old apiary | 4.12 | 3.93 | 3.95 |
|  |  |  |  |
| Infected | I REPETITION | II REPETITION | III REPETITION |
| 1-day-old | 0.09 | 0.09 | 0.09 |
| 19-day-old lab | 0.85 | 0.78 | 0.82 |
| 19-day-old apiary | 3.72 | 3.89 | 3.39 |
| 28-day-old lab | 2.96 | 2.88 | 2.79 |
| 28-day-old apiary | 3.78 | 3.62 | 3.85 |

Phenoloxidase activity, August

| Healthy | I REPETITION | II REPETITION | III REPETITION |
| --- | --- | --- | --- |
| 1-day-old | 0.13 | 0.142 | 0.158 |
| 19-day-old lab | 0.326 | 0.34 | 0.348 |
| 19-day-old apiary | 0.548 | 0.868 | 0.564 |
| 28-day-old lab | 0.226 | 0.354 | 0.358 |
| 28-day-old apiary | 1.756 | 2.378 | 1.016 |
|  |  |  |  |
| Infected | I REPETITION | II REPETITION | III REPETITION |
| 1-day-old | 0.086 | 0.096 | 0.092 |
| 19-day-old lab | 0.554 | 0.826 | 0.976 |
| 19-day-old apiary | 2.628 | 2.71 | 2.758 |
| 28-day-old lab | 0.298 | 0.296 | 0.26 |
| 28-day-old apiary | 3.298 | 3.454 | 3.424 |

Phenoloxidase activity, September

| Healthy | I REPETITION | II REPETITION | III REPETITION |
| --- | --- | --- | --- |
| 1-day-old | 0.132 | 0.118 | 0.138 |
| 19-day-old lab | 1.602 | 2.098 | 1.686 |
| 19-day-old apiary | 3.23 | 3.398 | 3.19 |
| 28-day-old lab | 0.714 | 0.602 | 0.572 |
| 28-day-old apiary | 1.298 | 1.376 | 1.376 |
|  |  |  |  |
| Infected | I REPETITION | II REPETITION | III REPETITION |
| 1-day-old | 0.086 | 0.086 | 0.084 |
| 19-day-old lab | 0.36 | 0.404 | 0.732 |
| 19-day-old apiary | 3.086 | 3.198 | 3.288 |
| 28-day-old lab | 1.106 | 1.162 | 1.154 |
| 28-day-old apiary | 1.724 | 1.6 | 1.626 |

Oxidative stress, May

| Healthy | I REPETITION | II REPETITION | III REPETITION |
| --- | --- | --- | --- |
| 1-day-old | 5.16 | 5.21 | 5.25 |
| 19-day-old lab | 3.63 | 4.01 | 4.00 |
| 19-day-old apiary | 7.70 | 9.84 | 7.68 |
| 28-day-old lab | 4.36 | 4.55 | 4.67 |
| 28-day-old apiary | 6.51 | 6.88 | 6.56 |
|  |  |  |  |
| Infected | I REPETITION | II REPETITION | III REPETITION |
| 1-day-old | 5.70 | 6.23 | 6.21 |
| 19-day-old lab | 4.59 | 4.79 | 4.78 |
| 19-day-old apiary | 8.85 | 8.98 | 10.96 |
| 28-day-old lab | 3.42 | 3.91 | 4.57 |
| 28-day-old apiary | 7.39 | 7.72 | 7.78 |

Oxidative stress, June

| Healthy | I REPETITION | II REPETITION | III REPETITION |
| --- | --- | --- | --- |
| 1-day-old | 5.66 | 5.75 | 5.96 |
| 19-day-old lab | 5.16 | 5.19 | 5.06 |
| 19-day-old apiary | 8.27 | 8.66 | 8.37 |
| 28-day-old lab | 4.70 | 4.80 | 4.82 |
| 28-day-old apiary | 5.88 | 6.30 | 6.55 |
|  |  |  |  |
| Infected | I REPETITION | II REPETITION | III REPETITION |
| 1-day-old | 4.00 | 3.95 | 4.09 |
| 19-day-old lab | 4.71 | 4.83 | 4.78 |
| 19-day-old apiary | 8.85 | 8.98 | 9.14 |
| 28-day-old lab | 4.21 | 4.37 | 4.32 |
| 28-day-old apiary | 7.23 | 9.51 | 7.21 |

Oxidative stress, July

| Healthy | I REPETITION | II REPETITION | III REPETITION |
| --- | --- | --- | --- |
| 1-day-old | 6.99 | 7.06 | 6.93 |
| 19-day-old lab | 4.03 | 4.16 | 4.09 |
| 19-day-old apiary | 7.11 | 6.88 | 7.02 |
| 28-day-old lab | 3.95 | 3.63 | 3.63 |
| 28-day-old apiary | 2.22 | 2.47 | 2.66 |
|  |  |  |  |
| Infected | I REPETITION | II REPETITION | III REPETITION |
| 1-day-old | 7.40 | 7.33 | 7.39 |
| 19-day-old lab | 5.78 | 5.76 | 5.95 |
| 19-day-old apiary | 9.55 | 9.64 | 9.72 |
| 28-day-old lab | 6.64 | 6.58 | 6.48 |
| 28-day-old apiary | 3.44 | 3.56 | 3.05 |

Oxidative stress, August

| Healthy | I REPETITION | II REPETITION | III REPETITION |
| --- | --- | --- | --- |
| 1-day-old | 6.12 | 6.11 | 6.21 |
| 19-day-old lab | 6.63 | 6.66 | 6.99 |
| 19-day-old apiary | 7.57 | 6.68 | 6.90 |
| 28-day-old lab | 4.32 | 4.29 | 4.18 |
| 28-day-old apiary | 7.53 | 7.22 | 7.07 |
|  |  |  |  |
| Infected | I REPETITION | II REPETITION | III REPETITION |
| 1-day-old | 4.56 | 4.56 | 4.70 |
| 19-day-old lab | 5.01 | 5.35 | 5.21 |
| 19-day-old apiary | 7.28 | 7.05 | 7.28 |
| 28-day-old lab | 4.48 | 4.75 | 4.68 |
| 28-day-old apiary | 7.45 | 7.70 | 7.54 |

Oxidative stress, September

| **Healthy** | I REPETITION | II REPETITION | III REPETITION |
| --- | --- | --- | --- |
| 1-day-old | 5.97 | 5.92 | 5.77 |
| 19-day-old lab | 4.78 | 4.88 | 4.73 |
| 19-day-old apiary | 6.72 | 4.94 | 5.41 |
| 28-day-old lab | 4.75 | 4.91 | 4.94 |
| 28-day-old apiary | 9.11 | 8.71 | 9.27 |
|  |  |  |  |
| **Infected** | I REPETITION | II REPETITION | III REPETITION |
| 1-day-old | 5.46 | 5.39 | 5.56 |
| 19-day-old lab | 6.85 | 4.71 | 5.90 |
| 19-day-old apiary | 6.76 | 6.74 | 7.00 |
| 28-day-old lab | 5.10 | 5.32 | 5.20 |
| 28-day-old apiary | 9.41 | 9.78 | 9.61 |

**Original gels**





**Original gel S1 Fig.**


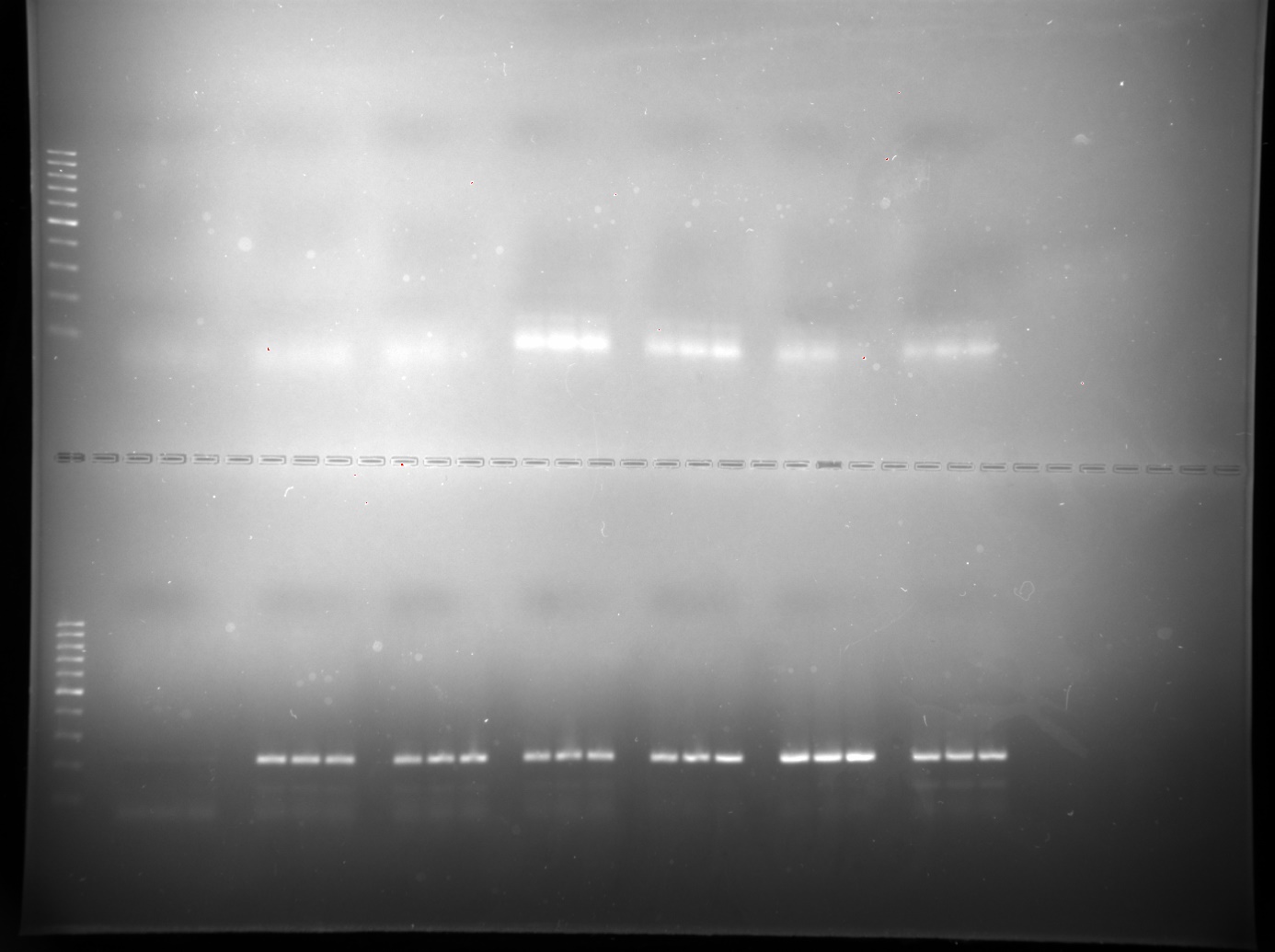


**Original gel S2 Fig.**





**Original gel S3 Fig.**


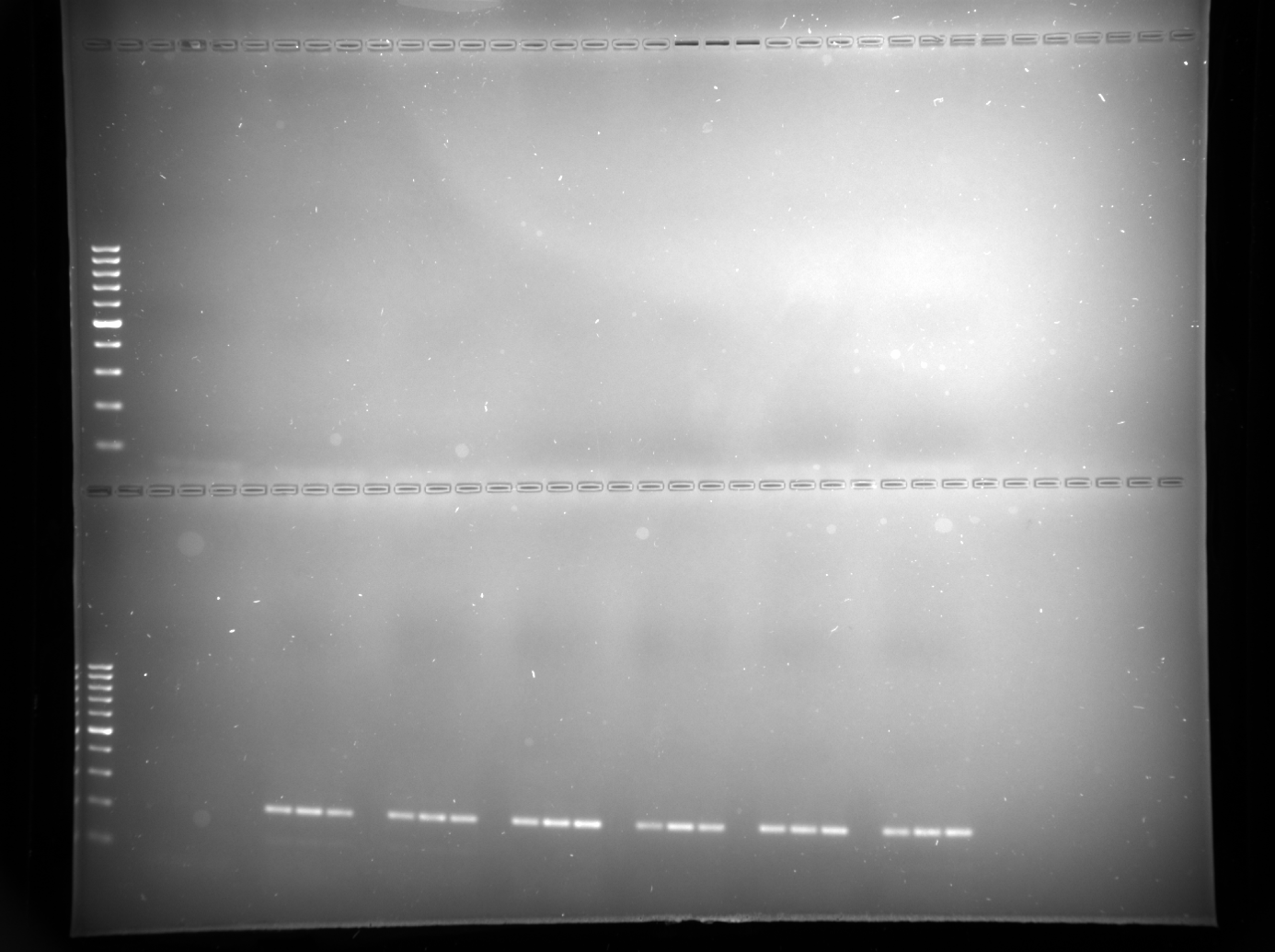


**Original gel S4 Fig.**


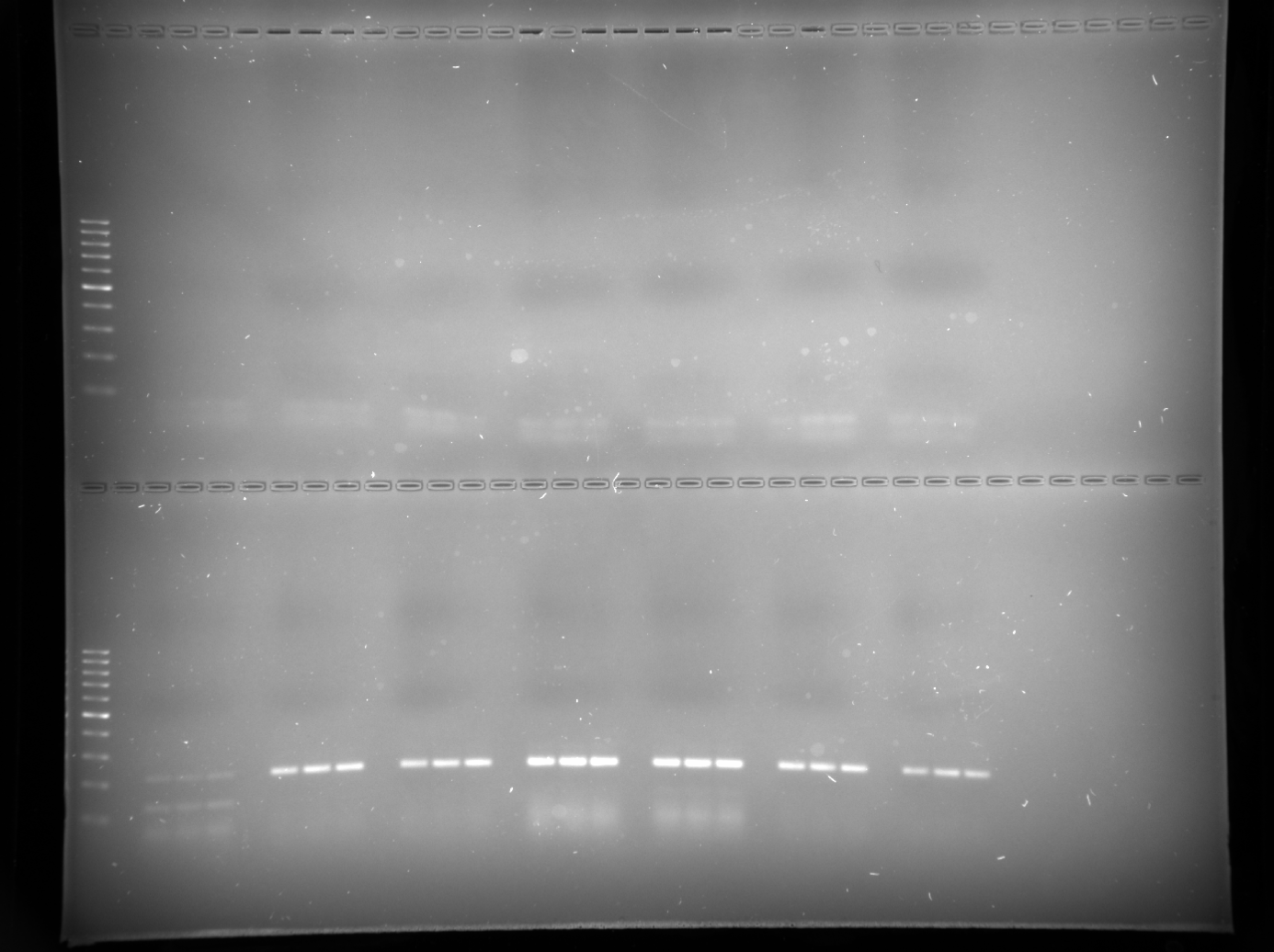


**Original gel S5 Fig.**
