# Supplementary figures and images for: Changes in enzymatic activity and oxidative stress in honeybees kept in the apiary and laboratory conditions during the course of nosemosis
Source: PLoS One. 2025 Jan 15;20(1):e0317384. doi: 10.1371/journal.pone.0317384 (PMC11734893; doi:10.1371/journal.pone.0317384)

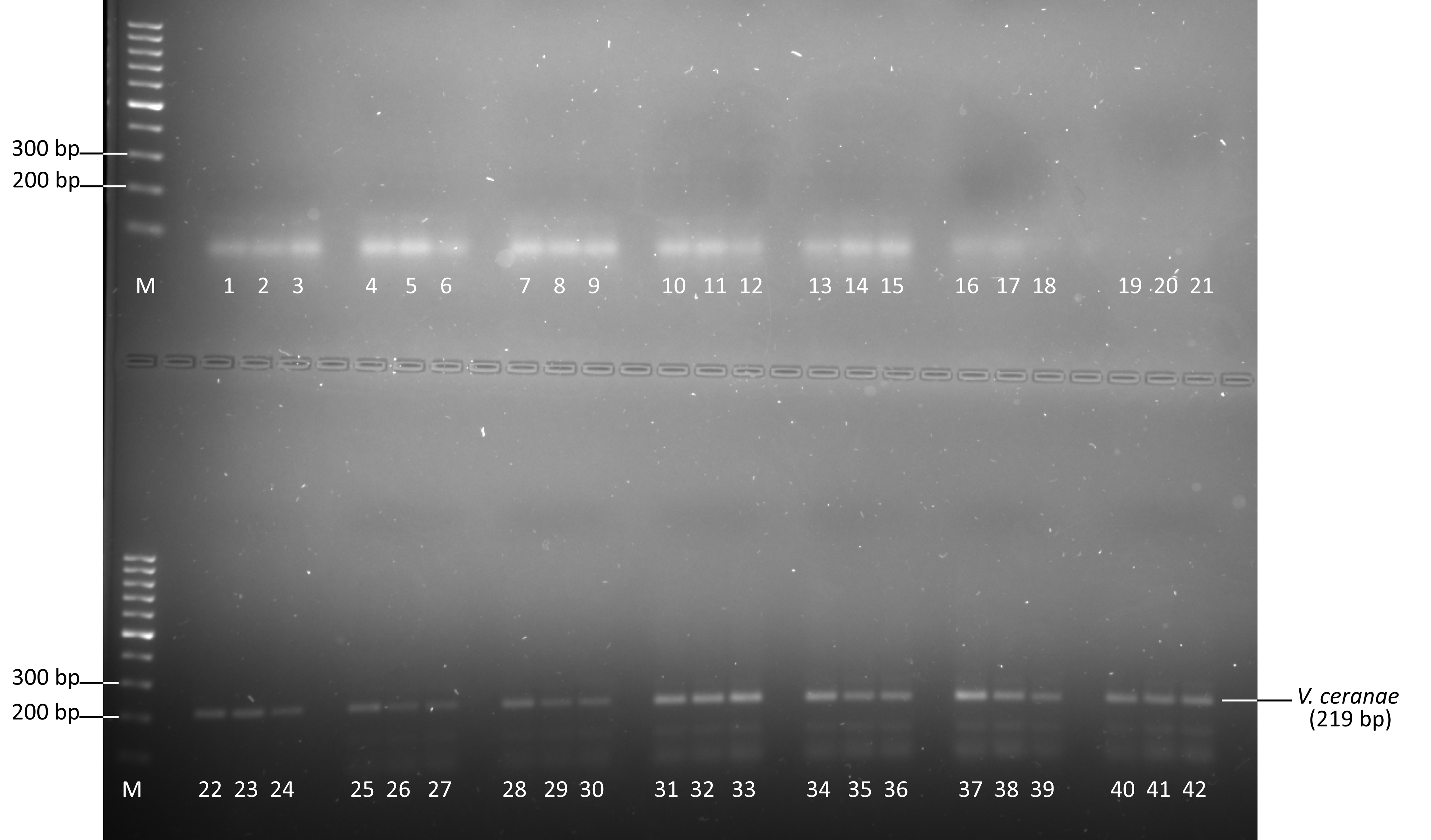

Supplement: S1 Fig — The designations of the samples of honeybees are shown in S1 Table. M–DNA Ladder (100 bp). (TIF) [file pone.0317384.s003.tif]

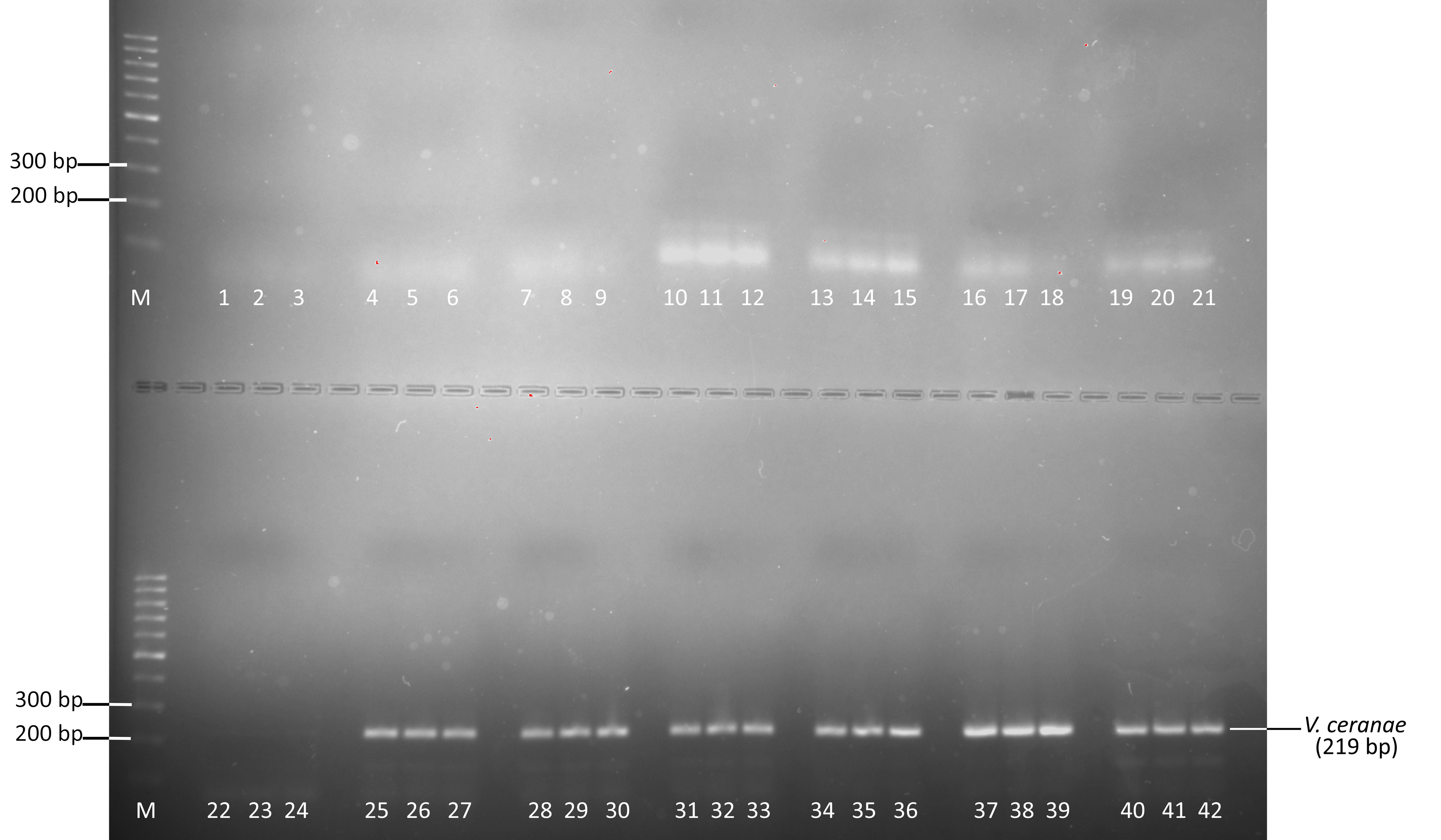

Supplement: S2 Fig — The designations of the samples of honeybees are shown in S1 Table. M–DNA Ladder (100 bp). (TIF) [file pone.0317384.s004.tif]

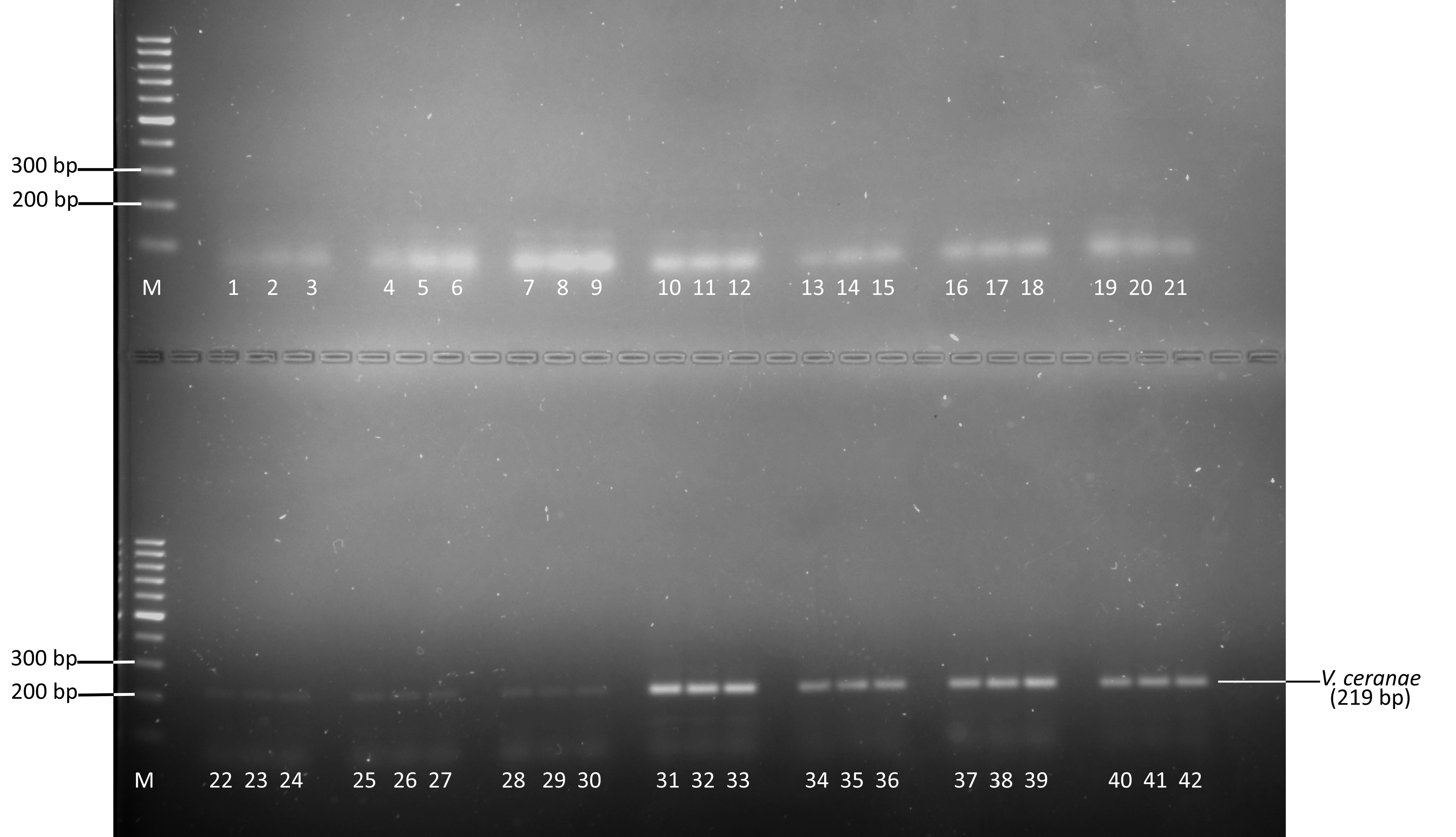

Supplement: S3 Fig — The designations of the samples of honeybees are shown in S1 Table. M–DNA Ladder (100 bp). (TIF) [file pone.0317384.s005.tif]

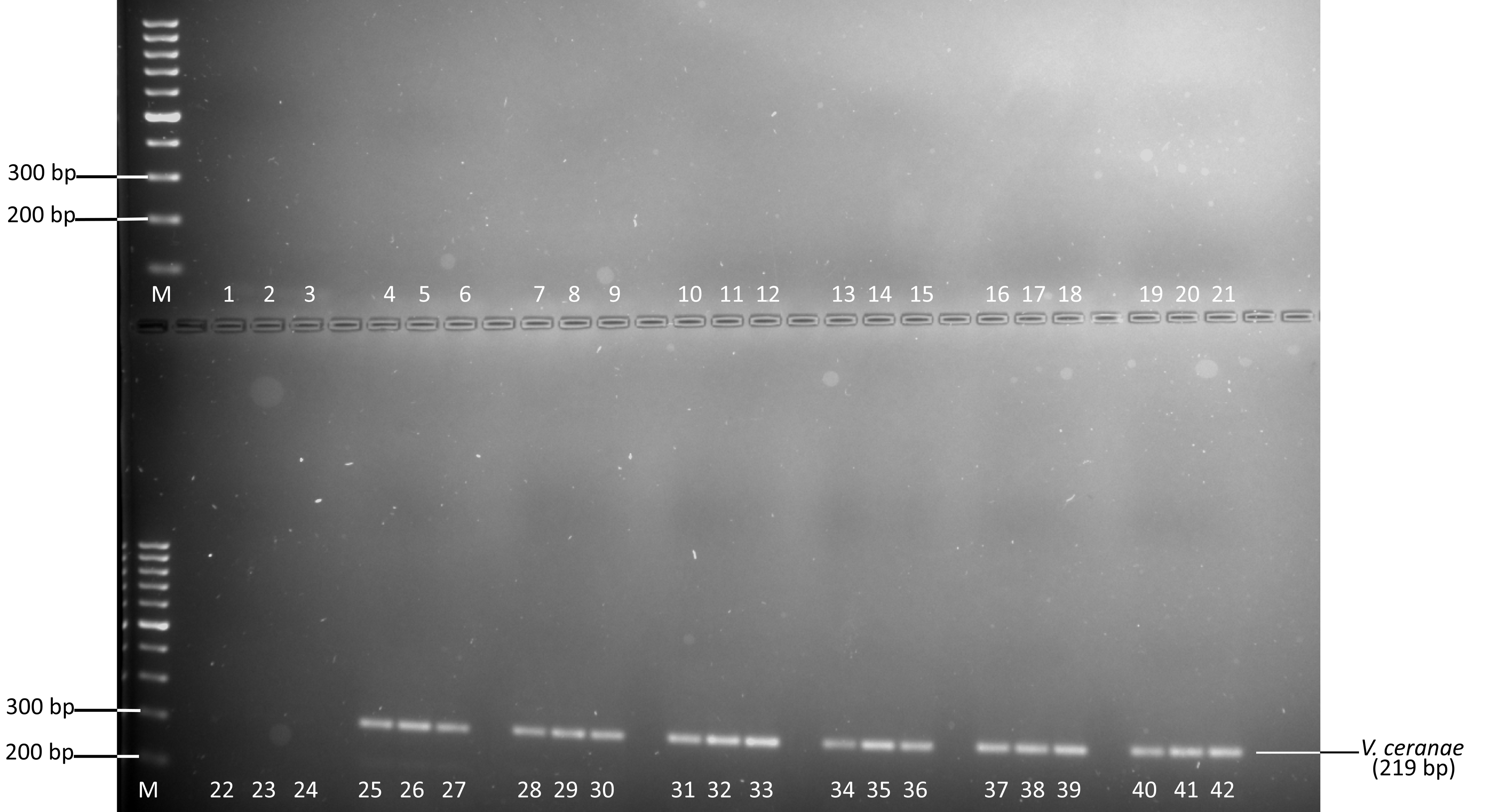

Supplement: S4 Fig — The designations of the samples of honeybees are shown in S1 Table. M–DNA Ladder (100 bp). (TIF) [file pone.0317384.s006.tif]

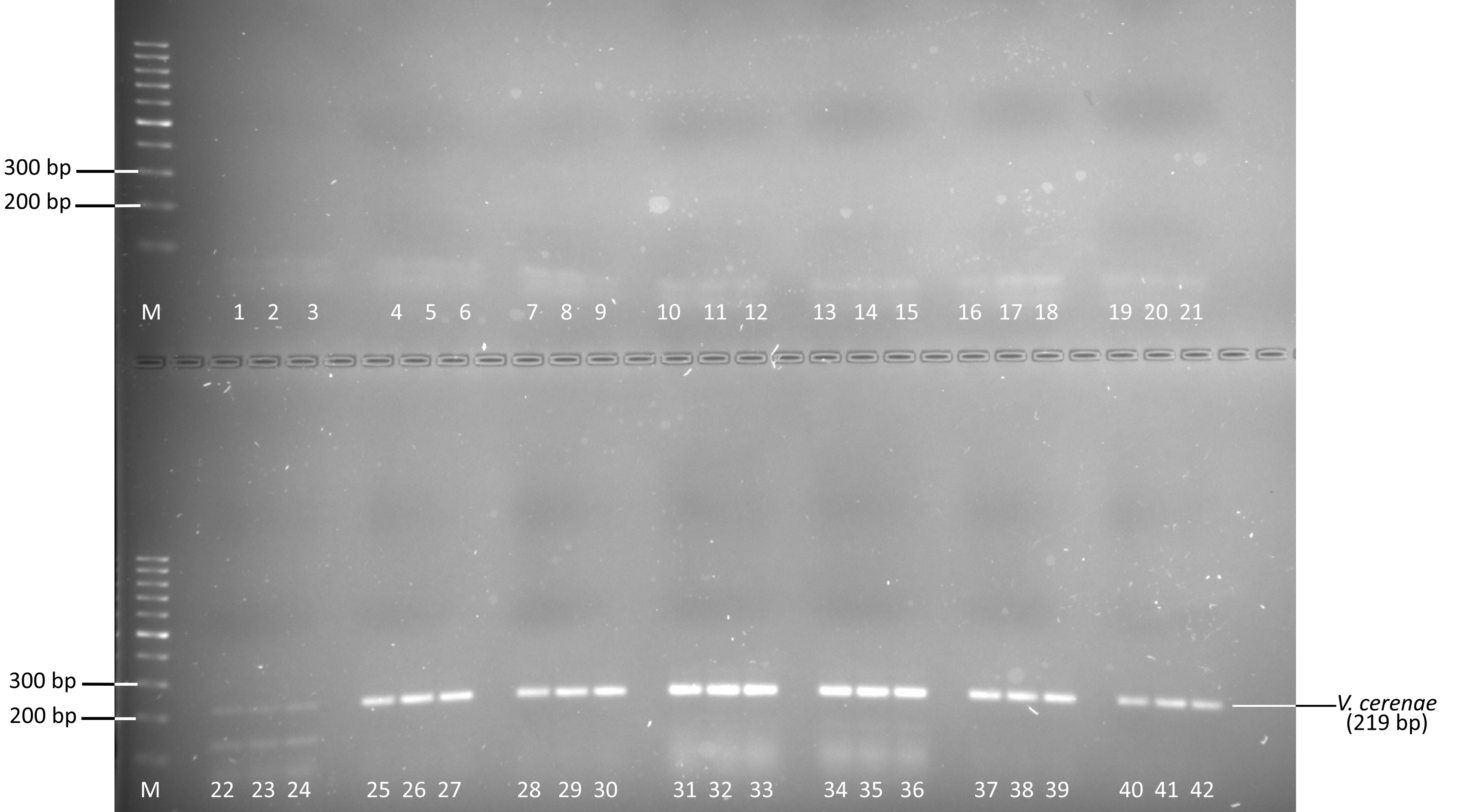

Supplement: S5 Fig — The designations of the samples of honeybees are shown in S1 Table. M–DNA Ladder (100 bp).; Raw data, Originals gels. (TIF) [file pone.0317384.s007.tif]
